# Antibody-based redirection of universal Fabrack-CAR T cells selectively kill antigen bearing tumor cells

**DOI:** 10.1136/jitc-2021-003752

**Published:** 2022-06-21

**Authors:** Yi-Chiu Kuo, Cheng-Fu Kuo, Kurt Jenkins, Alfur Fu-Hsin Hung, Wen-Chung Chang, Miso Park, Brenda Aguilar, Renate Starr, Jonathan Hibbard, Christine Brown, John C Williams

**Affiliations:** 1 Department of Molecular Medicine, City of Hope National Medical Center, Duarte, California, USA; 2 Department of Hematology and Hematopoietic Cell Transplantation, City of Hope National Medical Center, Duarte, California, USA; 3 Irell and Manella Graduate School of Biological Sciences, City of Hope National Medical Center, Duarte, California, USA

**Keywords:** immunotherapy, immunotherapy, adoptive, receptors, chimeric antigen, cell engineering

## Abstract

**Background:**

Chimeric antigen receptor (CAR) T cells engineered to recognize and target tumor associated antigens have made a profound impact on the quality of life for many patients with cancer. However, tumor heterogeneity and intratumoral immune suppression reduce the efficacy of this approach, allowing for tumor cells devoid of the target antigen to seed disease recurrence. Here, we address the complexity of tumor heterogeneity by developing a universal CAR.

**Method:**

We constructed a universal Fabrack-CAR with an extracellular domain composed of the non-tumor targeted, cyclic, twelve residue meditope peptide that binds specifically to an engineered binding pocket within the Fab arm of monoclonal antibodies (mAbs). As this site is readily grafted onto therapeutic mAbs, the antigen specificity of these universal Fabrack-CAR T cells is simply conferred by administering mAbs with specificity to the heterogeneous tumor.

**Results:**

Using in vitro and in vivo studies with multiple meditope-engineered mAbs, we show the feasibility, specificity, and robustness of this approach. These studies demonstrate antigen- and antibody-specific T cell activation, proliferation, and IFNγ production, selective killing of target cells in a mixed population, and tumor regression in animal models.

**Conclusion:**

Collectively, these findings support the feasibility of this universal Fabrack-CAR T cell approach and provide the rationale for future clinical use in cancer immunotherapy.

**WHAT IS ALREADY KNOWN ON THIS TOPIC**

- Universal CAR systems with split CAR designs have been developed to increase the flexibility of antigen recognition and to improve controllability of CAR effector function.

**WHAT THIS STUDY ADDS**

- This study has developed a novel universal CAR platform using the meditope technology. These universal fabrack-CAR T cells incorporate the meditope peptide as the tumor targeting domain and when combined with meditope-enabled antibodies display highly specific and potent antitumor activity against a broad range of tumor targets.

**HOW THIS STUDY MIGHT AFFECT RESEARCH, PRACTICE AND/OR POLICY**

- Fabrack CAR T cells combined with meditope-enabled antibodies may be a therapeutic strategy to overcome antigen escape and to improve safety of conventional CAR T cells.

## Introduction

As evidenced by remarkable success in treating hematological malignancies, where the majority of patients achieved complete tumor response in the relapsed and refractory setting, adoptive T cell therapy using chimeric antigen receptor (CAR) T cells is a potent approach to treat cancer.^1–3^ Given this success, there are multiple efforts to expand this therapy to additional hematological cancers as well as to solid tumors.^4^ And while the success of CAR T cell therapy is clear, its application across multiple cancers has also exposed important limitations,^5^ which include relapse due to antigen escape, and potentially life-threatening adverse events, including on-target, off-tumor toxicities, cytokine release syndrome (CRS), and immune effector cell-associated neurotoxicity syndrome.^6^ Likewise, this highly bespoke therapy requires the generation of unique CAR designs for each clinical setting, which incurs significant costs in terms of time and money. As such, there are a number of challenges that need to be addressed in order to realize the broad application of cellular therapies to treat disease.

To address antigen escape of conventional CAR T cells and minimize the occurrence of side effects, a number of groups have developed universal CAR systems.^7–9^ The underlying basis of this approach is to replace the CAR tumor targeting domain with a unique receptor that does not directly recognize malignant cells, but instead specifically recognizes an antigen targeting molecule. This flexible design allows for a variety of adaptors recognizing a broad spectrum of antigens on target cells without re-engineering T cells. These adaptors can be given sequentially or in combination to address antigen heterogeneity. Likewise, the concentration and affinity of an adaptor can be modified to specifically target diseased cells. They can also be adjusted to avoid hyperactivation of the immune system and enable more fine-tuned control of therapeutic activity for improved safety.

Here, we use meditope technology, discovered and developed in our lab, to create a CAR that is universal for all cancers. Based on diffraction studies, we identified a cyclic, 12 amino acid peptide bound to a site that lies between the light and heavy chains of cetuximab.^10^ We demonstrated that the residues that line this site are unique to the murine-chimeric cetuximab. While the residues needed to bind to the cyclic peptide are absent in human monoclonal antibodies (mAbs), we have demonstrated that we can readily graft this site onto human mAbs to enable peptide binding.^11^ Due to the position of this site on the Fab, we have termed the peptide a meditope, and mAbs bearing the grafted residues as meditope-enabled mAbs (memAbs).^10^ Through a series of biophysical studies, we have markedly improved the affinity of the interaction and developed this system to conjugate memAbs with imaging agents, toxins, and other biologics, either through mechanically interlocked meditopes^12^ or through template-catalyzed disulfide functionalization.^13^

To create a universal CAR T cell using this meditope technology, we have simply replaced the antigen recognition domain with a meditope peptide and used memAbs to define the specificity. Akin to adding a bike rack to a car to carry one or more bikes to a specific site, we have coined the meditope-bearing ‘receptor’ as a Fabrack. We have characterized the composition of the Fabrack-CAR (eg, linker and transactivation sequences), used multiple meditope-enabled Fabs, characterized the activation (eg, CD107a and IFNγ expression) as a function of antigen density, demonstrated that combinations of memAbs with different antigen specificities target antigen presenting cells, and demonstrated in vivo that the Fabrack-CAR T cell eliminated tumor xenografts individually or as a combination. Collectively, these studies suggest the feasibility of universal CAR T cells using Fabrack and support further development for clinical use in cancer immunotherapy.

## Materials and methods

### Cell lines and reagents

Human breast cancer cell lines SKBR3 (ATCC HTB-30), BT474 (ATCC HTB-20), MCF7 (ATCC HTB-22), and MDA-MB-468 (ATCC HTB-132) were cultured in Dulbecco′s Modified Eagle Medium (DMEM) (Corning #10013CV). Human ovarian cancer cell line OVCAR3 (ATCC HTB-161) was cultured in RPMI (Corning #10040CV) and SKOV3 was cultured in DMEM. Human chronic myeloid leukemia cell line K562 (ATCC CCL-243) and acute myeloid leukemia cell lines MV411 (ATCC CRL-9591) and HL60 (ATCC-CCL-240) were cultured in RPMI. Jurkat-NFAT-Luc cells (Invivogen #jktl-nfat) were cultured in RPMI. Human embryonic kidney (HEK) cell line 293T (ATCC CRL-3216) was cultured in DMEM. All media were supplemented with 10% (v/v) fetal bovine serum (FBS) (Omega Scientific #FB-11), 100 U/mL penicillin, and 100 µg/mL streptomycin (Corning #30 002 CI). Cells were maintained at 37°C with 5% CO_2_ in a humidified incubator. Chinese hamster ovary (CHO)-S cells (Gibco #R8007) were cultured in CHO Expression Medium (Gibco #12651014) in spinner flasks on an orbital shaker and maintained at 37°C with 5% CO_2_ in a humidified incubator. NFAT: nuclear factor of activated T-cells.

### Antibody production

Antibodies were produced by transient transfection of ExpiCHO cells (Gibco) based on the manufacturer’s high titer protocol. To purify the antibodies, ExpiCHO medium was centrifuged (12,000 × g, 30 min, 4°C), followed by passage through 0.45 micron and 0.22 micron filters. The clarified medium was then applied to protein G resin (GenScript), rinsed with 20 column volumes of phosphate buffered saline (PBS), and eluted with 10 column volumes of 100 mM glycine buffer, pH 3.0. Eluted antibodies were immediately neutralized with 1 M Tris, pH 9.0. Antibodies were further purified by size exclusion chromatography on an S200 26/60 (GE Healthcare) and stored in PBS at 4°C. For Fab purification, ExpiCHO medium was clarified and then purified using protein G resin as stated above. Monomeric Fabs were further purified by size exclusion chromatography using an S75 26/60 (GE Healthcare).

### Transient transfection of CHO-S cells

CHO-S cells were transiently transfected with no vector (Mock), HER2 scFv, Fabrack-CAR (CD28), and Fabrack-CAR (CH3-CD28) by the FreeStyle MAX transfection kit (Invitrogen) according to the manufacturer’s protocol.

### Lentivirus production for stable transfection

HEK293T cells were transfected with pCHGP2, pCMV-rev2, pCMV-G, and the lentiviral vectors with an interested gene to produce lentivirus. After 72 hours post-transfection, medium of HEK293T was collected and passed through a 0.45 micron filter. Clarified medium and Lenti-X (Takara #631231) were mixed at 3:1 ratio and incubated at 4°C for 30 min. The mixture was centrifuged at 1500 x g at 4°C for 45 min. After supernatant was removed, the virus pellet was resuspended in PBS and then stored in aliquots in −80°C freezer.

### DNA construct

The candidates of the Fabrack-CAR cassette contained a CSF2RA signal peptide (UniProtKB # P15509, 1–22 AA), an extracellular domain (ECD) composed of the non-tumor targeted, cyclic, 12 residue meditope peptide (CQFDLSTRRLQC), a linker (proline, alanine, and serine (PAS) linker sequence in our CAR construct is SAPASSASAPSAASAPA), with or without the CH3 domain of IgG4 heavy chain (GQPREPQVYTLPPSQEEMTKNQVSLTCLVKGFYPSDIAVEWESNGQ PENNYKTTPPVLDSDGSFFLYSRLTVDKSRWQEGNVFSCSVMHEALHNHYTQKSLSLSLGK), and a CD28 transmembrane domain followed by a cytoplasmic domain consisting of either a CD28/CD3ζ or 41BB/CD3ζ signaling sequence. HER2 scFv CAR cassette was composed of HER2 scFv, CH3 spacer (UniProtKB # P01860, 271-377 AA), CD28 transmembrane domain, CD28 costimulatory and CD3ζ cytolytic signaling sequences. The T2A ribosomal skip sequence was used to split the expression of the CAR and truncated CD19 (CD19t). The CD33-dsRed cassette was composed of a CD33 fragment Met1-Thr299, followed by a T2A ribosomal skip sequence and dsRed. Promoter EF1α in an epHIV7 lentiviral backbone was used to control the expression of the constructs.

### Fluorophore labeling of soluble proteins

Alexa Fluor dyes (Invitrogen) were attached to soluble proteins using amine conjugation according to the manufacturer’s protocol. Briefly, Alexa Fluor 647 NHS Ester dye was conjugated to HER2 memAb IgG and Fab and an CTLA4 IgG, ipilimumab. Degree of label (DOL) was calculated, using A280 and Amax, to be between 1≤DOL ≤ 3 dye per molecule. Pacific Blue NHS Ester dye was conjugated to the HER2 ECD (UniProtKB # P04626, 1-630AA). The method of manufacturing and purifying His-tagged HER2 ECD was previously described.^10^ Protein interactions were characterized by size-exclusion chromatography (SEC) prior to flow cytometry analysis to assess binding activity.

### Flow cytometry analysis of extracellular or intracellular protein

For general cell surface staining, cells were washed with 2% BSA in PBS at 400 x g at 4°C for 5 min. Cells were treated with primary antibody with or without fluorescent conjugates at 4°C for 30 min in the dark and washed. If a second staining was needed, cells were treated with secondary antibody at 4°C for 30 min in the dark and washed. Cells at 10^6^ cells/mL were analyzed by flow cytometry. The secondary antibodies were anti-kappa-Alexa-647 (Abcam #ab202832), anti-IgG Fc-488 (Invitrogen #H10120), and anti-IgG Fc-PE (Abcam #ab98596). For intracellular staining, cells were fixed and permeabilized by Fixation/Permeabilization Kit (BD #554715) according to the manufacturer’s protocol and the above stated staining procedure was performed.

### Native T cell functional assays

To analyze CD107a and IFN expression, cancer cells (5×10^4^/100 µL) were seeded in a 96-well round-bottom plate and human Mock or Fabrack T cells (5×10^4^/100 µL) were added to each well with existing cancer cells at a 1:1 E:T ratio. CD107a-fluorescein isothiocyanate (FITC) (BD #555800) antibody and transporter inhibitor Golgistop (BD #554724) were added to each well during incubation. After 5 hour incubation, cells were stained with fixable viability dye (Invitrogen #L34965) at 4°C for 30 min in the dark. After being washed twice, cells were stained with CD3 (BD #347347) and CD19-PECy7 (BD #557835) at 4°C for 30 min in the dark. After being washed twice, cells were fixed and permeabilized by BD Cytofix/Cytoperm kit (BD #554714) followed by staining intracellular IFN by IFN-APC (BD #554702) at room temperature for 30 min in the dark. After being washed twice, cells were resuspended and then analyzed by flow cytometry.

For assessing the tumor killing effect of Fabrack T cells, cancer cells (2.5×10^4^/100 µL) were seeded in a 96-well round-bottom plate and human Mock or Fabrack T cells (6,250/100 µL) with or without a memAb were added to each well with existing cancer cells. After 72 hours incubation, cells were stained with CD3 (BD #347347) and CD19-PECy7 (BD #557835) at 4°C for 30 min. After being washed twice, cells were resuspended in 2% BSA in PBS with 0.1 µg/mL 4',6-diamidino-2-phenylindole (DAPI) (Sigma #D9542) and then analyzed by flow cytometry.

### Viability assay

Cancer cells (2.5×10^4^/100 µL) and human T cells (6,250/100 µL) were seeded in each well in a 96-well round-bottom plate in the presence or absence of antibody. After 72 hours incubation, cells were centrifuged at 250 x g for 5 min and 100 µL of media in each well was removed. Cell viability was examined based on the instruction of Promega CellTiter-Glo Luminescence kit. After 10 min incubation, 100 µL of mixture was moved to a white-wall 96-well plate and read by Biotek’s Synergy 4 multidetection microplate reader. Viability (%) = [Lum (cancer cells+T cells+antibody) – Lum (T cells+antibody) ]/[Lum (cancer cells+T mock cells) – Lum (T mock cells)] Lum: luminescence.

### Jurkat-NFAT-Luc activation assay

Jurkat-NFAT-Luc cells were transduced with Fabrack-CAR by lentivirus and CD19t was used to sort Fabrack-CAR positive cells out by the Aria II SORP. Cancer cells (2.5×10^4^/100 µL) were seeded in a 96-well white-wall plate. After cells were attached overnight, media in the plate was removed and Fabrack Jurkat-NFAT-Luc cells (1×10^5^/60 µL) with or without antibody were added to each well. Cells were incubated at 37°C for 6 hours followed by addition of 50 µL luciferase substrate (Invivogen #rep-qlc2) to each well. The luminescence was immediately read by Biotek’s Synergy 4 multidetection microplate reader.

### Primary human T cells isolation and culture

Isolation and culture of human PBMC has been previously described.^14 15^ Central memory T (Tcm) cells were enriched by negative selection of CD14, CD25, and CD45RA and positive selection of CD62L. For expansion, cells were maintained in complete X-VIVO media with 50 U/mL recombinant human IL2 (Novartis) and 0.5 ng/mL recombinant human IL15 (CellGenix).

### Lentiviral transduction of human T cells

T cells were stimulated with Dynabeads Human T-Expander CD3/CD28 (Invitrogen) at a 1:3 ratio (T cell:bead) overnight in X-VIVO-15 (Lonza) supplemented with 10% FBS, 2 mM L-glutamine, and IL2/IL15 [50 U/mL IL2 (Novartis), 0.5 ng/mL IL15 (CellGenix)]. Stimulated T cells were then transduced with lentiviral vector (M.O.I. of 1.0) encoding the Fabrack-CAR. Mock and CAR transduced T cells were cultured with indicated cytokines three times a week for 18 days before subsequent analyses. Efficiency of CAR transduction in human T cells was 40%–60% (online supplemental figure S6).

10.1136/jitc-2021-003752.supp1 Supplementary data

### Microscopy

An equal amount of CD33 expressing MDA-MB-468 cells (2×10^4^) and HER2 expressing MDA-MB-468 cells (2×10^4^) were seeded in an 8-well chamber slide for attachment overnight. The next day, 2×10^4^ Fabrack T cells and memAb were added in each well. A Zeiss Observer Z1 microscope was used to capture live cell images every 10 min for 24 hours.

### Cell area measurement by ImageJ

Since live adherent cells can attach to slides and show broader cell area than dead detached cells, area of live HER2 or CD33 positive cells was quantified by setting the GFP or DsRed threshold to at least read the area of attached cells by ImageJ. An image was split into a red or green channel in order to measure GFP or DsRed-expressing cells in each image. The threshold was set to pick the area of GFP or DsRed-expressing cells without including background (Image >Adjust>Threshold). Chosen area was read by clicking Analyze >Measure. Cell area at 0 hour was set as 100%.

### Xenograft mouse model

Female NSG mice were intraperitoneally injected with firefly luciferase (Luc) engineered OVCAR3 cells (5×10^6^) on day one and randomly divided into eight groups (n=6 each group) on day 4. Antibodies were intraperitoneally injected into mice at a dose of 1.25 mg/kg every 3 days starting at day 4. Fabrack-CAR T cells (10×10^6^) were intraperitoneally injected once on day 5. To detect tumor burden in mice, 150 mg/kg D-Luciferin (PerkinElmer #122799) was intraperitoneally injected into mice and bioluminescence intensity was measured by the Lago X Imaging System. Mice were euthanized when abdominal distension, rough hair coat, body weight loss, or sickness was found. Kaplan-Meier survival curves were plotted by GraphPad Prism.

### Statistical analysis

Data are presented as mean±SEM, unless otherwise stated. Figures were generated using GraphPad Prism, while statistical analyses were performed using R. The non-parametric Mann-Whitney-Wilcoxon test was used to find statistically significant differences between experimental treatments/conditions, when four or more replicates were available per group. When fewer replicates were present, to increase power, data from each such experiment was analyzed by an analysis of variance (ANOVA), with Tukey’s test of Honest Significant Difference being used to find statistically significant differences between the experimental treatments/conditions. ANOVA assumptions were verified by means of Bartlett’s tests, and both visual analysis of residuals, as well as Shapiro-Wilks tests of normality of the residual distribution. When appropriate the data was transformed (eg, logarithmically) before ANOVA to ensure both lack of heteroskedasticity, and also a reasonable expectation of normally distributed measurement errors over the given experiment.

## Results

### Generation and characterization of Fabrack-CAR

**Figure 1:**
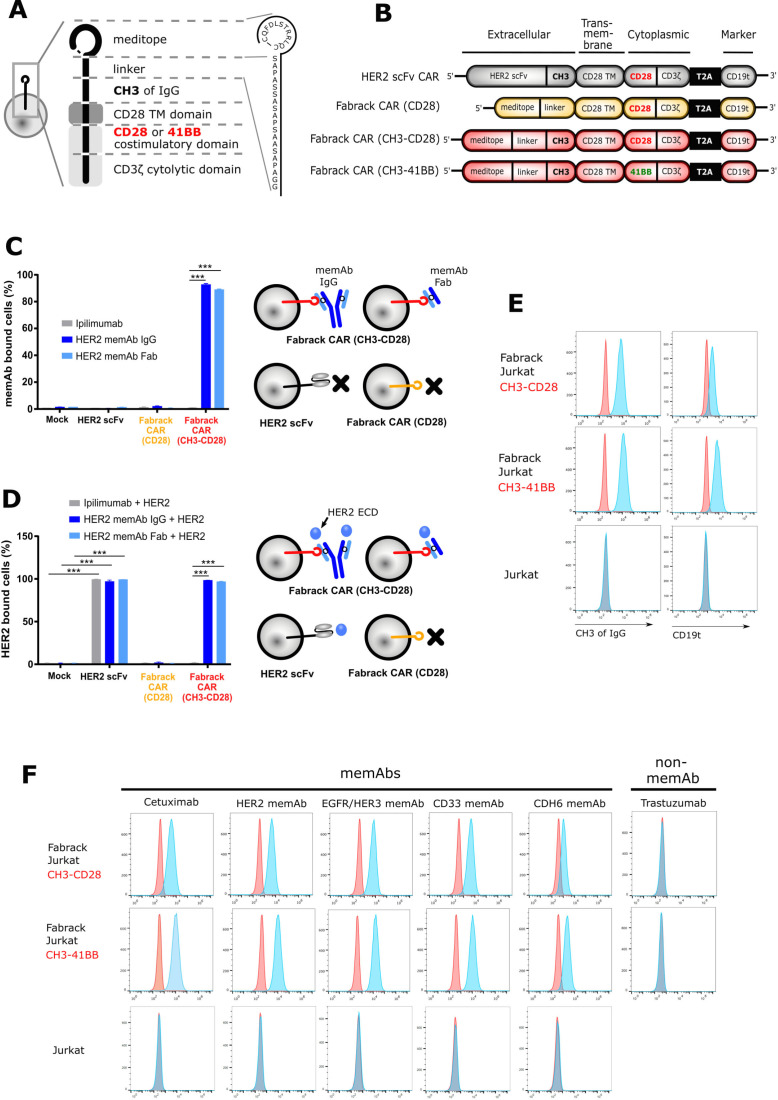
Generation and characterization of Fabrack-CAR for universal CAR T cell platform. (A) Schematic representation of protein structure of Fabrack-CAR on a cell. Amino acid sequence of meditope and linker: CQFDLSTRRLQC-SAPASSASAPSAASAPA. (B) Schematic representation of the lentiviral expression cassette of conventional HER2 scFv CAR and candidates of Fabrack-CAR, which incorporated an extracellular domain with or without CH3 spacer, a CD28 transmembrane (TM) domain, and a cytoplasmic region comprizing either CD28/CD3ζ, or 41BB/CD3ζ signaling domains for T cell signaling. A truncated nonsignaling CD19 (CD19t) following a T2A ribosomal skip sequence was expressed separately from CAR construct for tracking successfully transduced cells. (C) The ability of Fabrack-CAR binding to memAb or meFab. CHO-S cells transfected with HER2 scFv CAR or candidates of Fabrack-CAR were examined for their binding to αHER2 memAb and meFab conjugated with Alexa-647 by flow cytometry, with gating on CD19t+cells. Ipilimumab, an αCTLA-4 non-memAb, was used as a negative control (mean±SEM, ***p<0.001). (D) HER2-binding ability of HER2 scFv CAR, αHER2 memAb, or meFab-coupled meditope-CAR. The binding of HER2 extracellular domain (ECD)-Pacific Blue to cells were examined by flow cytometry. Experiments were done in technical triplicates (mean±SEM, ***p≤0.001). (E) Validation of Fabrack expression on Jurkat cells. Fabrack positive cells were confirmed by staining cells with αFc-PE or αCD19-PECy7 antibody followed by flow cytometry. Two independent experiments were done. (F) Validation of multiple memAbs binding to Fabrack Jurkat cells by flow cytometry. The binding of memAbs to Fabrack Jurkat cells was analyzed by flow cytometry after cells were stained with secondary anti-human kappa-Alexa-647. MemAbs only bound to Fabrack Jurkat cells, whereas Jurkat cells without Fabrack expression did not have memAb binding. All data are representative of at least two independent experiments. CAR, chimeric antigen receptor; memAbs, meditope-enabled monoclonal antibodies.

To generate proof of concept that the meditope functions as a cellular ‘Fabrack’, the scFv of a conventional CAR was replaced with the meditope peptide and a linker while conserving the CD28 transmembrane, CD28 costimulatory, and CD3 signaling domains (figure 1A, B). In addition, we designed two Fabrack-CAR constructs with different extracellular spacers. The short spacer was composed of a 17-amino acid-PAS linker only,^16^ while the long spacer was constructed of the PAS linker and the CH3 domain of IgG4. Also included in the CAR cassette was a truncated CD19 (CD19t) for transgene expression, which was separated from the CAR by a T2A ribosomal skip sequence.^14^ For initial characterization of the Fabrack-CAR constructs, each variant was transiently expressed on CHO-S cells and flow cytometry was used to confirm successful transfection by detecting CD19t expression on the cell surface (online supplemental figure S1). HER2 scFv CAR was used as a control as it has been well studied.^14^ These data show only cells transfected with the CH3-CD28 construct were capable of αHER2 memAb IgG and Fab binding (figure 1C). The Fabrack-CAR lacking a CH3 domain did not produce a detectable signal. This observation could be due to low expression, inaccessibility, or rapid turnover of the construct on the surface of the cell membrane. Flow cytometry showed that meditope-CH3-CD28 bearing cells, decorated with either αHER2 memAb or meFab, bind the HER2 ECD at comparable levels to the scFv-HER2-CAR controls (figure 1D). Ipilimumab, an αCTLA4 mAb which is not meditope-enabled and should not bind to cells transduced with Fabrack CAR, was used as a negative control. These results confirmed the expression of Fabrack CAR (CH3-CD28) on the cell membrane, as illustrated in figure 1A, and this meditope functioned as a rack for memAbs and Fabs.

Establishing the proof-of-concept, both meditope-CH3-CD28 and meditope-CH3-41BB Fabrack constructs were individually expressed in Jurkat cells by lentiviral transduction to create stable cell lines. Expression of the Fabrack was once again confirmed by staining cells with αFc and αCD19 antibodies, which bound to the CH3 domain within the CAR spacer and truncated CD19, respectively (figure 1E). Next, flow cytometry confirmed the ability of the engineered Fabrack Jurkat cells, either with CD28 or 41BB co-stimulatory domains, to bind to memAbs using cetuximab, αHER2, αEGFR/HER3, αCD33, and αCDH6 memAbs.^10 17–19^ As negative controls, clinical trastuzumab (eg, not meditope-enabled) did not bind the Fabrack T cells, and parental Jurkat cells did not bind memAbs (figure 1F, bottom row). The mutation sites for memAbs are shown in online supplemental figure S2.

**Figure 2:**
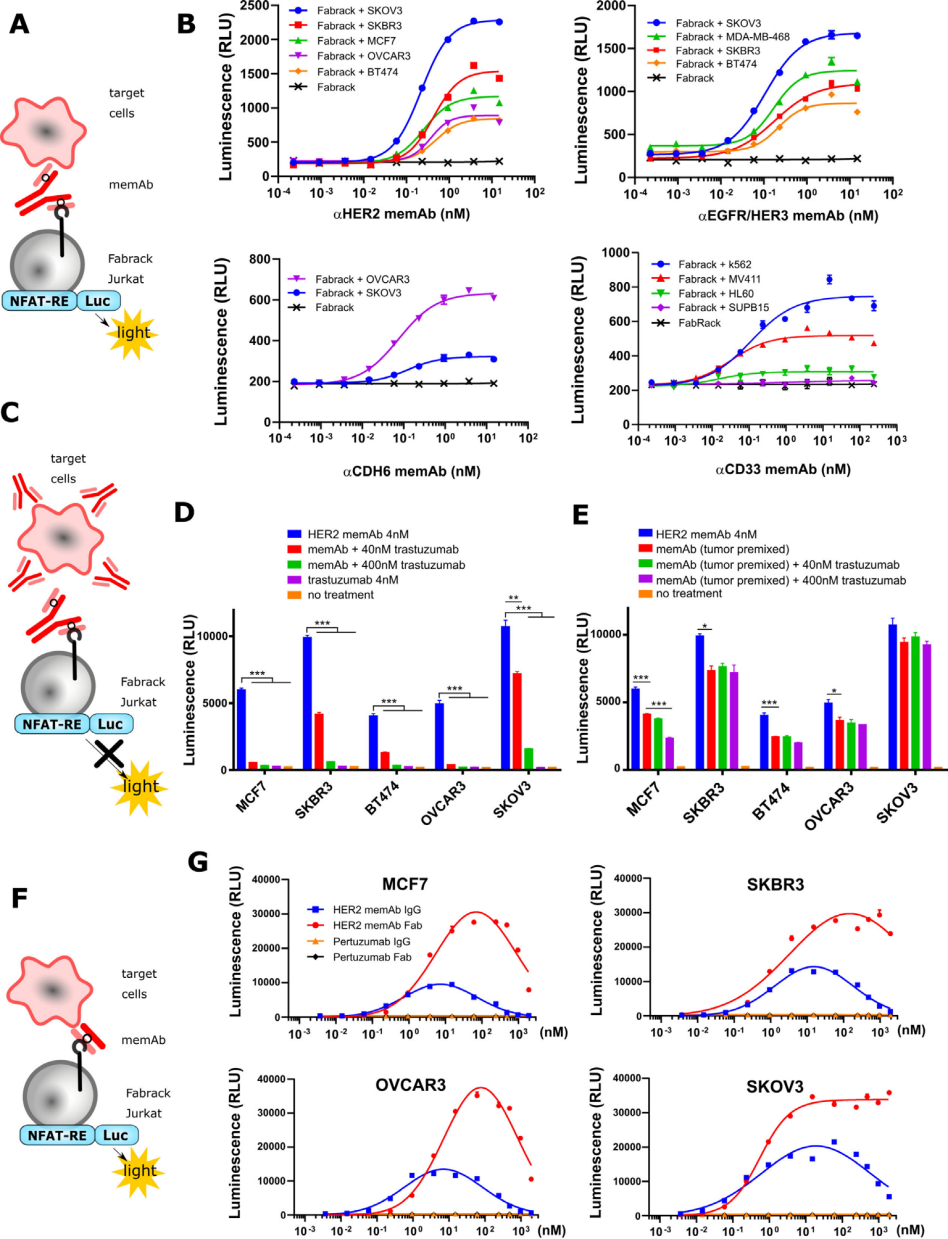
Activation of Fabrack Jurkat cells in the presence of target cells and a memAb. Fabrack with CH3-CD28 construct was examined in this figure. (A) NFAT regulated luciferase in Fabrack Jurkat cells was used as an index to measure T cell activity. NFAT-RE: NFAT-response element. (B) MemAb-mediated concentration-dependent activation of Fabrack Jurkat cells. Four-fold serial dilution of αHER2 memAb (upper left), αEGFR/HER3 memAb (upper right), αCDH6 memAb (lower left), and αCD33 memAb (lower right) was prepared to redirect Fabrack Jurkat cells to target cells. After 6 hour incubation, luciferase substrate was added in each well and luminescence was measured immediately. The data are representative of two independent experiments (mean±SEM). (C) Illustration showing that antibody without a meditope-enabled site blocked memAb-mediated Fabrack Jurkat cell activation. (D) Clinical trastuzumab dose-dependently blocked αHER2 memAb-mediated Fabrack Jurkat cell activation. Trastuzumab at 40 nM (red bar) or 400 nM (green bar) was used to block 4 nM αHER2 memAb-mediated activation of Fabrack Jurkat cells. During incubation, HER2 memAb and clinical trastuzumab were present simultaneously. After 6 hours incubation, luciferase substrate was added in each well and luminescence was measured immediately. Experiments were done in technical duplicates. Significance versus blue bar is indicated (mean±SEM, **p< 0.01, ***p< 0.001). (E) Clinical trastuzumab slightly blocked HER2 memAb-mediated Fabrack Jurkat cell activation when target cells were premixed with 100 nM HER2 memAb and washed out. Experiments were done in technical duplicates. Significance versus red bar is indicated (mean±SEM, *p<0.05, ***p<0.001). (F) Illustration shows that meFab binding to both Fabrack Jurkat cells and target cells. (G) Activation of Fabrack Jurkat cells was mediated by αHER2 memAb or meFab in the presence of HER2 expressing breast (MCF7 and SKBR3) or ovarian (OVCAR3 and SKOV3) cancer cell lines. Non-meditope-enabled anti-HER2 pertuzumab IgG or Fab were used as negative controls. All data are representative of at least two independent experiments (mean±SEM). NFAT-RE: NFAT-response element. memAb, meditope-enabled monoclonal antibody.

### Activation of Fabrack Jurkat cells based on meditope-enabled antibody targeting

After establishing distinct memAbs binding to the transformed cells and their respective antigen simultaneously, we transformed the Jurkat cells harboring an NFAT responsive luciferase gene (Jurkat-NFAT-Luc) with the Fabrack construct to characterize the relationship between the activation of T cell signaling pathways and the presence of tumor-associated antigen bearing cells and the antigen-specific memAbs (figure 2A). Using this system, we observed that activation of these luciferase expressing cells was dependent on antigen bearing cells, and the level of activation was memAb concentration dependent (figure 2B and online supplemental figure S3). The level of antigen recognition for each memAb to each cell line was determined by flow cytometry and, where possible, compared with the commercial/clinical equivalent mAb (online supplemental figure S4). Median fluorescent intensity showing relative antigen level on the cell surface is reported in online supplemental tables S1 and S2. Of note, the target cells with higher antigen expression typically induced higher activation of Fabrack Jurkat cells. Interestingly, BT474, which has high HER2 expression, produced much lower luminescence than that of low HER2-expressing cell lines, such as OVCAR3 and MCF7. This phenomenon was also observed in high CD33 expressing HL60 cells. Further characterization of these cell lines is needed to understand these observations.

**Figure 3:**
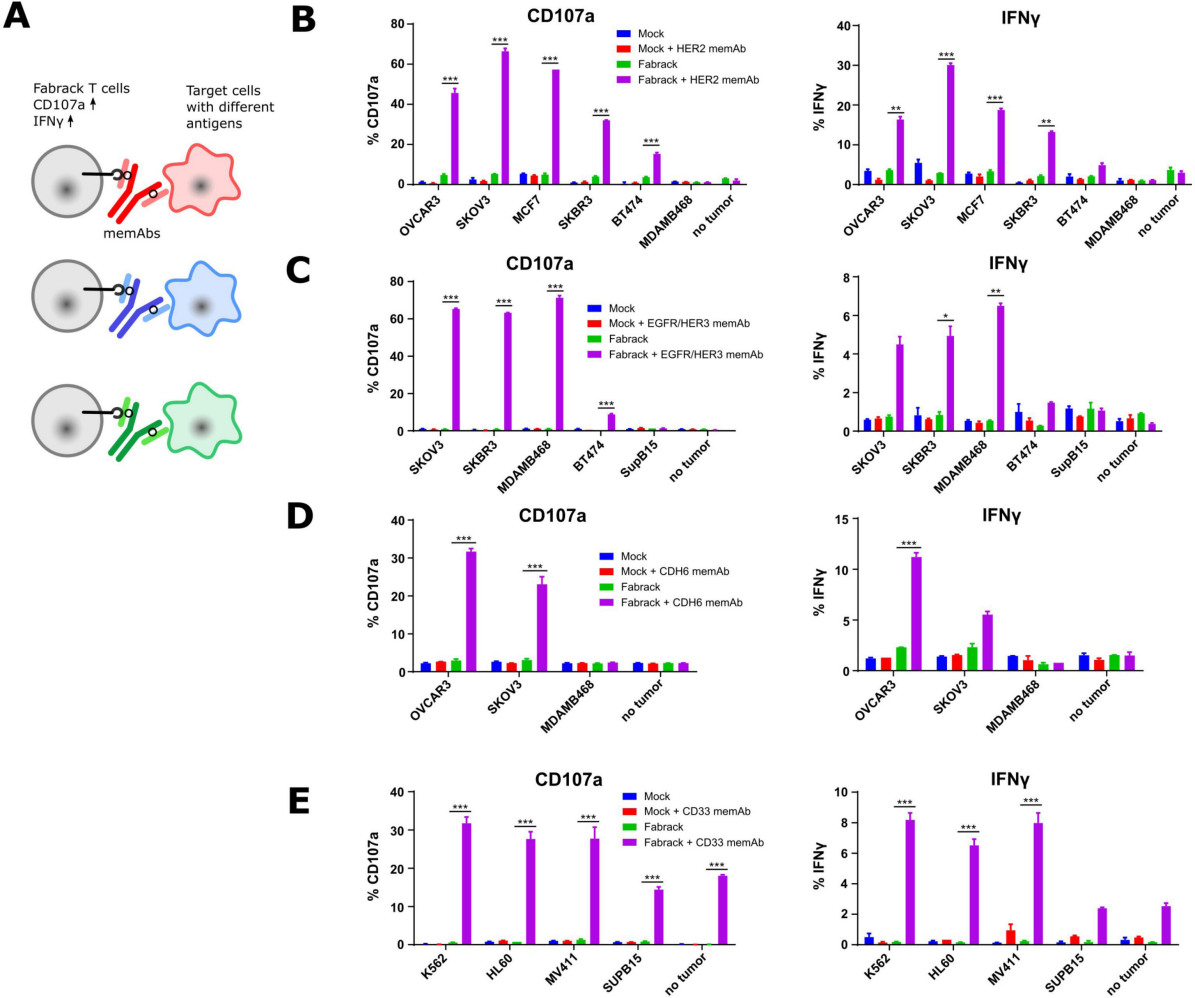
Activation of Fabrack T cells in the presence of target cells and a corresponding memAb. (A) Diagram showing the increased activation markers, CD107a and IFNγ, of Fabrack T cells after redirection of Fabrack T cells to target cells by a memAb. (B–E) The percentage of T cells with increased CD107a or IFNγ is shown. Activation of Fabrack T cells was observed (purple bars). Cells were incubated with or without 0.5 nM αHER2 memAb. Mock was gated on all CD3 +cells and Fabrack conditions were gated on CD19+(CAR+) cells. (B), αEGFR/HER3 memAb (C), αCDH6 memAb (D), or αCD33 memAb (E) for 5 hours at a 1:1 E:T ratio. Increased expression of CD107a (left) and IFNγ (right) in Fabrack T cells was analyzed by flow cytometry after staining cells with fluorescent dye-conjugated antibodies. Experiments were done in technical duplicates (mean±SEM, *p<0.05, **p<0.01, ***p<0.001). CAR, chimeric antigen receptor; memAb, meditope-enabled monoclonal antibody.

To further confirm antigen specificity, we used clinical trastuzumab (which is not meditope-enabled and, thus, incapable of binding to the Fabrack Jurkat cells) to test whether activation of the Fabrack-NFAT-Luc Jurkat cells could be blocked and whether the order of addition affects the activation (figure 2C). First, we observed that the clinical trastuzumab blocked αHER2, trastuzumab-based memAb Fabrack Jurkat cell activation in a concentration-dependent manner (figure 2D). Second, we observed that the activation of the Fabrack-NFAT-Luc Jurkat cells first treated with αHER2 memAb, washed, and then incubated with clinical trastuzumab (again, not meditope-enabled) produced a slight reduction in the overall activation compared with Fabrack-NFAT-Luc Jurkat mixed with αHER2 memAb and directly mixed with antigen bearing cells (figure 2E). These observations were consistent in breast cancer cell lines (MCF7, SKBR3, and BT474) and ovarian cancer cell lines (OVCAR3 and SKOV3) (figure 2D, E).

Finally, we observed the hook or prozone effect at high concentration of memAbs.^20^ This effect is ascribed to the saturation of target cells and Fabrack T cells with the memAb. To further characterize this observation, we produced the meFab of the αHER2, trastuzumab-based memAb to reduce the valency and, thus, overall affinity to HER2 (figure 2F). We extended the titration of the meFab and memAb to higher concentrations (eg, to 1 µM) across four cell lines (figure 2G). In all cases for the mAb, the luminescence increased to ~10 nM and then decreased until effectively absent at 1 µM. For the meFabs, the peak luminescence occurred at 100 nM, except for the SKOV3 cell lines where the luminescence reached a maximum at ~10 nM and remained at that level to the highest concentration. Flow cytometry analysis of CAR expression demonstrates that the luminescence decrease was not due to antibody-induced CAR internalization (online supplemental figure S5). Moreover, in all cases, we observed a higher level of luminescence using meFabs compared with memAbs. These observations likely reflect differences in affinity and/or internalization due to valency (eg, bivalent memAbs can cluster and drive internalization).^21–23^

### Activation of Fabrack-engineered human T cells is meditope-antibody dependent

Based on the proof-of-concept activation studies in Jurkat cells, we proceeded to evaluate the Fabrack platform in primary human T cells engineered to express the non-targeting Fabrack-CAR (online supplemental figure S4). First, we examined memAb-dependent upregulation of the activation marker CD107a and production of inflammatory cytokine IFNγ as a measure of cytolytic activity of the transduced Fabrack-CAR (CD28-CH3) human T cells (figure 3A). These early markers of T cell activation were measured following 5 hours co-culture of T cells and target cells at the ratio of 1:1 with or without a memAb. The results showed mock T cells exhibited negligible background activity with or without a memAb, while Fabrack T cells showed robust activation in the presence of target cells and a corresponding memAb (figure 3B–E). In accordance with NFAT-regulated Jurkat cell activation, SKOV3 induced the highest activation of T cells at 0.5 nM αHER2 memAb and BT474 induced lowest activation (figure 3B). Likewise, when incubated with αEGFR/HER3 memAb, BT474 also showed lowest activation, both in Fabrack Jurkat and native Fabrack T cells (figure 3C). With respect to CDH6, OVCAR3 cells with higher CDH6 expression elicited stronger T cell activation than SKOV3 cells (figure 3D). As for αCD33 memAb, different target cells showed a similar activation level of T cells, although Fabrack Jurkat cell activation was quite different between K562, HL60, and MV411 (figure 3E). We also observed activation of Fabrack T cells by CD33 memAb in the absence of targeted tumor cells. Low levels of CD33 expression on native T cells likely accounts for this observation (online supplemental figure S7).^24^ Importantly, tumor cells that did not express the antigen targeted by specified memAb did not activate Fabrack T cells, again, suggesting the specificity of memAb binding to an antigen and the meditope peptide. In addition to CD107a and IFNγ, increased expression of CD25 and CD69 was also detected on Fabrack T cells dependent on αHER2 memAb and HER2 presenting target cells (online supplemental figure S8). These data demonstrated that memAbs linking to both target cells and Fabrack T cells allowed the functional activation of human, Fabrack-CAR T cells.

### Tumor killing ability of Fabrack T cells

**Figure 4:**
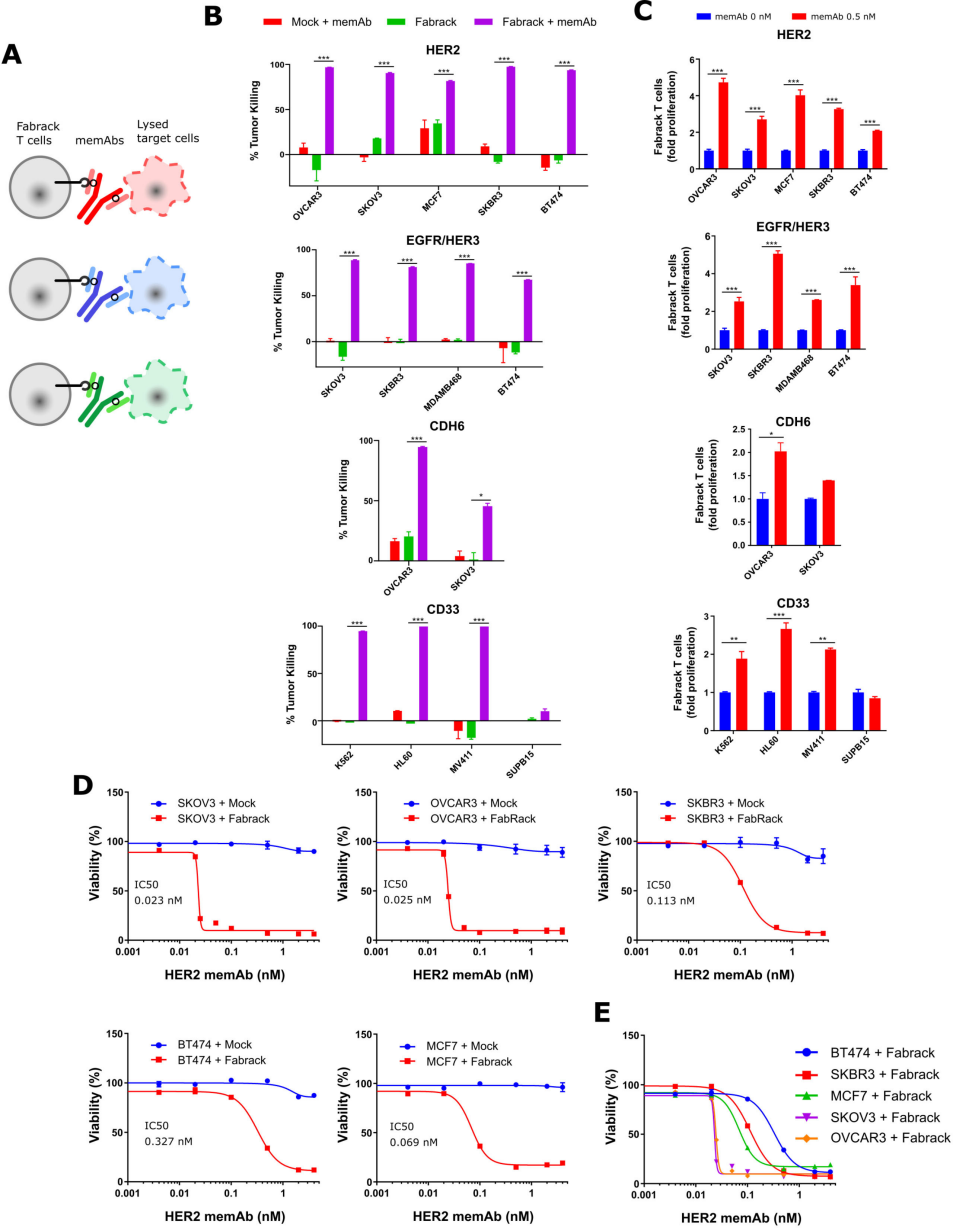
Tumor killing by Fabrack T cells accompanied with T cell proliferation. (A) Diagram showing tumor killing by Fabrack-CAR T cells after redirection of T cell by a memAb. (B) Tumor-killing ability of Fabrack T cells is represented by a purple bar. Cells were incubated for 3 days at a 1:4 E:T ratio with or without 0.5 nM αHER2 memAb, αEGFR/HER3 memAb, αCDH6 memAb, or αCD33 memAb administered at the beginning of culture for 3 days at a 1:4 E:T ratio. The killing of indicated cancer cells was analyzed by flow cytometry after DAPI staining. Killing was based on tumor counts co-cocultured with Mock T cells. Experiments were done in technical duplicates (mean±SEM, *p<0.05, ***p<0.001). (C) Proliferation of Fabrack T cells was analyzed by flow cytometry by gating CD3 and CD19 double positive cells after incubation of T cells with indicated target cells and a memAb for 3 days. Experiments were done in technical duplicates (mean±SEM, *p<0.05, **p<0.01, ***p<0.001). (D) The tumor-killing ability of Fabrack T cells was dependent on memAb concentration. The viability and IC50 of each cancer cell line is shown. The indicated cancer cells were co-cultured with mock (blue) or Fabrack (red) T cells at a 1:4 E:T ratio in the presence of different concentrations of αHER2 memAb administered at the beginning of culture for 3 days. At the end of incubation, cell viability was measured based on the Promega Cell Titer kit instructions. Experiments were done in technical duplicates (mean±SEM). (E) The viability of each cancer cell line obtained from figure (D) was combined in the same plot. CAR, chimeric antigen receptor; memAb, meditope-enabled monoclonal antibody.

The ability of Fabrack T cells to kill antigen expressing tumor cells was investigated (figure 4A). We co-cultured Fabrack T cells and target cells at a ratio of 1:4 for 3 days and examined the lysis of target cells. As shown in figure 4B, C, αHER2, αEGFR/HER3, αCDH6, and αCD33 memAbs at 0.5 nM directed robust tumor killing by Fabrack T cells which was also accompanied with increased T cell population. Whereas Fabrack T cells coupled with 0.5 nM memAb killed at least 80% of the tumor population in most cancer cell lines, less than 50% killing was observed in SKOV3 cells treated with Fabrack T cells combined with the αCDH6 memAb. The reduced levels likely reflect the low CDH6 antigen expression on SKOV3.

In addition, we examined tumor killing and T cell proliferation induced by HER2 memAb vs meFab in different conditions—that is, with ET ratios at 1:1, 1:2, or 1:4 and incubation times of 24, 48, or 72 hours (online supplemental figure S9). Fabrack T cells directed by HER2 meFab attained similar or higher tumor killing than Fabrack T cells directed by HER2 memAb.

To determine whether the activation level of the Fabrack-CAR T cells can be controlled by mAb concentration, tumor and Fabrack T cells were co-cultured at various concentrations of memAb and the cell viability was determined. The viability of target cells was concentration dependent, giving rise to IC50 of αHER2 memAb-induced Fabrack T cell killing at 0.023 nM (SKOV3), 0.025 nM (OVCAR3), 0.069 nM (MCF7), 0.11 nM (SKBR3) and 0.33 nM (BT474) (figure 4D, E). The nearly 10-fold difference in the IC50 between these cell lines may partially reflect the different CD107a and IFNγ levels described above. In addition, the sudden decrease observed in SKOV3 and OVCAR3 viability starting at ~0.02 nM implies their higher susceptibility to T cell-mediated killing.^25 26^

### Fabrack T cells for heterogeneous tumor targeting

**Figure 5:**
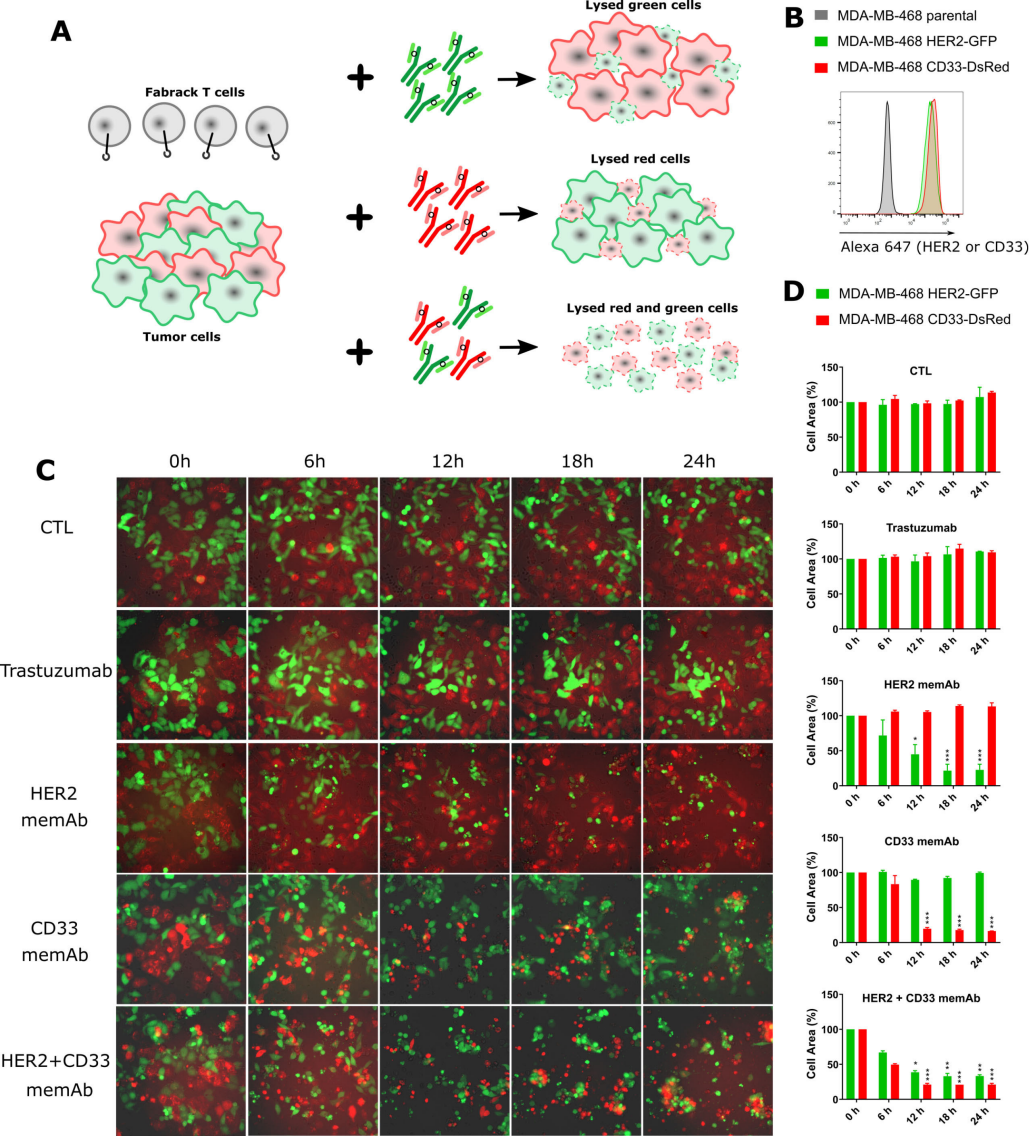
Specific tumor killing by Fabrack T cells in a heterogeneous MDA-MB-468 tumor. (A) Diagram showing mAb-specific tumor killing by Fabrack T cells. (B) Validation of HER2 or CD33 expression at the cell surface of MDA-MB-468 cells by flow cytometry. (C) Cell images were captured at various time points by an Observer Z1 time-lapse microscope. MDA-MB-468 cells with HER2-GFP expression were killed by Fabrack T cells in the presence of αHER2 memAb, while MDA-MB-468 cells with CD33t-DsRed expression were killed in the presence of CD33 memAb. Images are representative of two different spots under the microscope. (D) Histograms showed the percentage change of area of HER2-GFP positive or CD33-dsRed positive cells in captured images. Cell area of two different spots was quantified by ImageJ to obtain mean and SEM values. Each treatment at 0 hour was set as 100% for normalization. Experiments were done in technical duplicates. Significance versus time point at 0 hour is indicated (mean±SEM, *p<0.05, **p<0.01, ***p<0.001). memAb, meditope-enabled monoclonal antibodies. CTL: control condition without antibody treatment.

Based on the specific activation and tumor killing ability of Fabrack T cells shown in previous experiments, we asked whether we could selectively kill tumor cells in a heterogeneous environment using individual or a combination of memAbs (figure 5A). To establish a heterogeneous mixture of tumor cells, HER2-GFP or CD33-DsRed was introduced by lentiviral transduction to HER2/CD33 negative MDA-MB-468 cells (figure 5B). Expression of each was confirmed by flow cytometry. Fabrack T cells combined with antigen-specific memAb were introduced to a co-culture of HER2-GFP and CD33-DsRed expressing cells. Cell lysis was observed using fluorescence microscopy over a 24-hour period (figure 5C). In the absence of antibody or in the presence of clinical trastuzumab (eg, not meditope enabled), both HER2 and CD33 expressing cells remained intact, indicating the absence of cytotoxicity. However, the presence αHER2 memAb led to specific lysis of HER2-GFP expressing cells by Fabrack T cells, but not the CD33-DsRed expressing cells. A similar phenomenon was observed in the condition treated with αCD33 memAb. Moreover, the presence of both αHER2 and αCD33 memAbs lead to lysis of both target cells. A decrease in HER2 or CD33 positive cells reflects cell lysis of HER2 or CD33 expressing cells (figure 5D). In addition, we used HER2 and EGFR/HER3 memAbs to treat a heterogeneous population of MDA-MB-468 and Raji-HER2-GFP cells and demonstrated selective killing throughflow cytometry analysis (online supplemental figure S10). Taken together, these cell-based assays suggest that the Fabrack T cells coupled with multiple memAbs recognizing unique antigens could eradicate a heterogeneous tumor.

### Tumor elimination in vivo using Fabrack T cells coupled with memAbs

**Figure 6:**
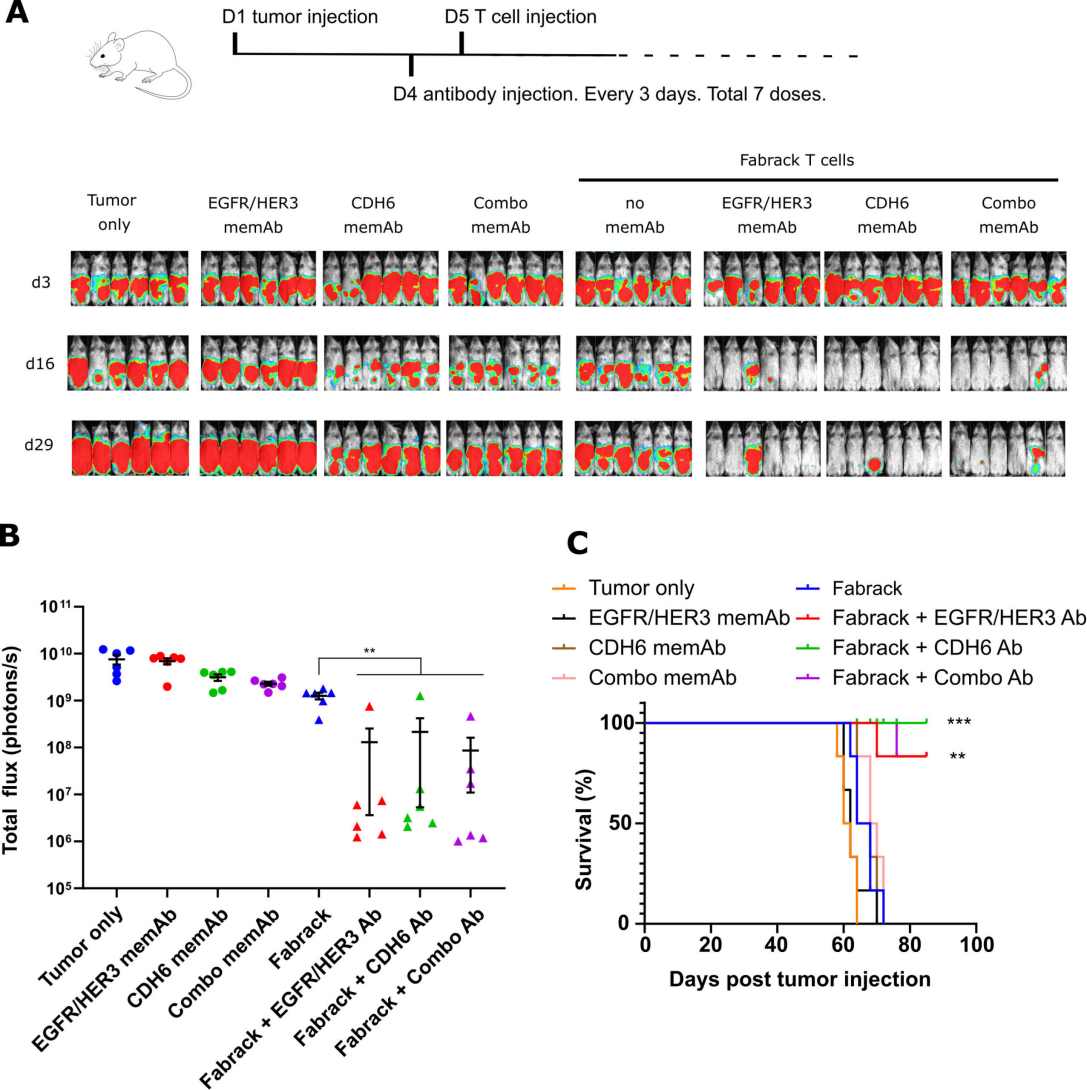
In vivo activity of Fabrack T cells in an OVCAR3 xenograft model. (A) Mouse images of OVCAR3 tumor burden were shown on days 3, 16, and 29. Mice were divided into eight groups for different treatments, including tumor only, memAb treatment at a dose of 1.25 mg/kg (αEGFR/HER3 memAb, αCDH6 memAb, and 0.625 mg/kg αEGFR/HER3 +0.625 mg/kg αCDH6 memAb (combo)), 10^7^ Fabrack T cell treatment, or 10^7^ Fabrack T cells in combination with indicated memAb. (B) Tumor burden of mice in different treatment groups was plotted based on total flux at day 29 (n=6, median ±SEM, **p<0.01). (C) Fabrack T cells in combination with indicated memAb demonstrated improved survival. Log rank test for the Kaplan Meier curves was used to calculate P values. Significance versus Fabrack T cell treated group is indicated (**p<0.01, ***p<0.001). memAb, meditope-enabled monoclonal antibody.

To test the efficacy of the Fabrack T cell system, we characterized tumor reduction using a xenograft model. To establish tumor grafts in mice, 5×10^6^ OVCAR3 cancer cells that stably express firefly luciferase (Luc) were injected intraperitoneally into immunodeficient NOD.Cg-Prkdcscid Il2rgtm1Wjl/SzJ (NSG) mice. Tumor growth in each mouse was tracked by in vivo imaging of bioluminescence activity from Luc-expressing OVCAR3 cells. In a pilot study, we treated mice with HER2 memAb at 0.25 and 1.25 mg/kg every 2 days for seven doses in combination with one dose of Fabrack T cells, and found that the dose of 1.25 mg/kg suppressed tumor better (online supplemental figure S11). Thus, we chose the dose of 1.25 mg/kg in the following studies. We further changed the Ab regimen to once every 3 days, since Luo *et al* demonstrated steady Ab concentrations in mouse plasma for up to 72 hours after intraperitoneal injection of cetuximab.^27^ As the OVCAR3 cells had high expression of EGFR/HER3 and CDH6 based on the antigen profile from flow cytometry (online supplemental figure S2), we used αEGFR/HER3 and αCDH6 memAbs to direct Fabrack T cell lysis of the OVCAR3 xenografts. The day prior to the injection of 10×10^6^ Fabrack T cells, the mice were treated intraperitoneally with 1.25 mg/kg of the individual memAb and then treated with the same dose every 3 days thereafter. We also tested a combination of the two memAbs, but both at half the concentration of the individual mAbs. Specifically, 0.625 mg/kg EGFR/HER3 memAb and 0.625 mg/kg CDH6 memAb were administered to the mice, again, following the same dosing schedule. Tumor burden was largely suppressed in mice treated with Fabrack T cells using either the individual or combination of memAbs (figure 6A, B). The control groups, treated with memAb alone or Fabrack T cells alone, showed minimal influence on tumor burden compared with the untreated group. Moreover, Fabrack T cells in combination with memAb extended the survival of mice compared with no treatment and either Fabrack T cells alone or Ab alone treatment (figure 6C). The animal study demonstrated the robust activity of Fabrack T cells to eradicate tumor cells in at least 4 of 6 mice in vivo when single memAb or multiple memAbs are used to redirect Fabrack T cells in target disease. We also examined different doses of Fabrack T cells (online supplemental figure S12) and found that an initial dose of 2×10^6^ and 10×10^6^ Fabrack T cells both provided a significant tumor suppression when combined with memAbs. However, a first dose of 10×10^6^ Fabrack T cells provided better tumor control than 2×10^6^ Fabrack T cells (both groups had a second dose of 2×10^6^ Fabrack T cells). Furthermore, on day 20, Fabrack T cells were detectable in the blood of mice treated with 10×10^6^ Fabrack T cells with or without memAb and, although administration of the memAb significantly reduced these systemic levels of cells, this is presumably due to the cells homing/trafficking to the tumor.

## Discussion

This study demonstrates that meditope technology can be used to create a universal CAR T cell platform by expressing Fabrack on T cells and administering different memAbs to target and lyse cells expressing specific antigens. As such, it potentially addresses a series of clinical issues associated with the current generation of CAR T cells, namely, antigen escape and CRS. Specifically, we demonstrate the killing capability of Fabrack-CAR using multiple memAbs specific to different antigens, including previously established αHER2 memAb and three new meditope-grafted memAbs, which targeted EGFR/HER3, CDH6, or CD33.^10^ Flow cytometry showed that these memAbs specifically bound to target cells and Fabrack T cells. In an MDA-MB-468 heterogeneous tumor comprizing HER2-GFP positive cells and CD33-DsRed cells, time-lapse images clearly demonstrated the specific tumor killing by Fabrack T cells in the presence of a corresponding memAb and capability of Fabrack T cells to eradicate a heterogeneous tumor. Finally, we demonstrated tumor reduction in vivo using memAbs, individually or in combination, that target unique tumor antigens.

We note other universal CAR T cell systems have been recently described such as FITC, a 14-aa peptide from yeast transcription factor GCN4, a 10-aa peptide form the human nuclear autoantigen La/SS-B, a protein motif of leucine zippers, and others.^7 9 28–33^ While the Fabrack system described here confers many of the same properties described in these systems, there are several major features that differentiate our approach from these, one of which is the compatibility with both Fabs and full length mAbs as bridging molecules. The potential advantage of a universal CAR platform that combines with full-length mAbs is multiple fold. First, mAbs themselves are therapeutic. Case in point, cetuximab, which naturally binds the meditope, is currently used in the clinic to treat head and neck and colorectal cancers.^34^ In addition to coupling Fabrack-CAR T cells to a tumor antigen, memAbs can retain their ability to bind NK and other immune cells and invoke favorable therapeutic processes such as antibody dependent cellular cytotoxicity and antibody-dependent cellular phagocytosis. Second, many antigens that have been and still are of intense interest to treat cancer are endogenous, but aberrantly overexpressed on tumors. The bivalent nature of mAbs also enhances their tumor selectivity based on avidity. Third, given the number of mAbs in the clinic, their pharmacokinetic and pharmacodynamic properties are well understood.^35^ As one of the features of a universal system, it is anticipated that additional doses of the antigen-CAR T cell bridging molecule will be administered weeks to even months later. The long half-life expected for molecules bearing an Fc allows the bridging moiety to find the tumor and the CAR T cells. Likewise, the size of an IgG reduces renal filtering. Fourth, mAbs are less likely to be immunogenic compared with other universal CAR T systems (eg, coiled coils), though, additional preclinical studies are needed to support this assertion. Finally, there are other practical benefits using mAbs for CAR T cell redirection compared with other bridging moieties. First, clinical mAbs are highly stable—often characterized with melting temperatures greater than 70°C and virtually no aggregation. Second, there is extensive experience in the manufacturing of mAbs. Third, there is extensive clinical experience in administering mAbs to patients. These practical benefits further reduce uncertainty using a novel therapeutic system.

While there are many potential advantages for using universal CAR T cells and a number of approaches being developed to leverage these advantages, there are limitations to universal CAR T cells. These include additional tumor-specific antigens, overcoming immune suppression, tumor penetration (eg, solid tumors), and immunogenicity of the targeting agents, all of which hold true for conventional CAR T cells. The most immediate concern of universal CAR T cells, however, is dosing, as evidenced by the hook effect observed at high concentrations of the universal antigen. This effect is driven by simultaneously saturating the antigen sites on the tumor and the universal CAR T cells which blocks the therapeutic effect. This hook effect, most widely recognized as the major driver for false negatives observed in ELISA-based diagnostics, is also a concern for other ‘bridging’ approaches including PROTACs.^36 37^ Further dosing studies will be needed to determine if and to what extent the hook effect affects the efficacy of this approach, however, there are approaches to address such issues. For instance, modulating the affinity of the interaction with the meditope/Fabrack, the affinity of the Fab to the target antigen, or both. By reducing the affinity, the dwell time on the Fabrack or antigen is reduced and, thus, when the tumor cell and Fabracked CAR T cell are juxtaposed, the universal moiety can simultaneously engage both. This approach mimics next generation bispecific immune engagers (eg, BiTEs) where the affinity of the anti-CD3 is reduced to improve the pharmacokinetics. Lowering the affinity of the Fab to the antigen also has the added advantage of increasing the specificity to antigens that are significantly overexpressed on tumor tissues (eg, HER2/EGFR). Consistent with this approach, most T-cell receptors (TCRs) bind major histocompatibility complex (MHC)-antigen complexes with low affinity. Other approaches are more involved but could include developing tumor-activated mAbs to reduce off-tumor toxicity (eg, tumor-activated mAbs).^38^

Overall, we demonstrate that universal, Fabrack-CAR T cells are a viable approach to address shortcomings of an otherwise potent therapeutic approach. Coupled with allogenic approaches being developed, we believe the use of fully functional mAbs provides unique advantages compared with other CAR T cell approaches, including universal CAR T cells, and merits further development to bring it to the clinic.

10.1136/jitc-2021-003752.supp2 Supplementary video

10.1136/jitc-2021-003752.supp3 Supplementary video

10.1136/jitc-2021-003752.supp4 Supplementary video

10.1136/jitc-2021-003752.supp5 Supplementary video

10.1136/jitc-2021-003752.supp6 Supplementary video

## Data Availability

Data are available in a public, open access repository.
