# Supplementary material for: Antibody-based redirection of universal Fabrack-CAR T cells selectively kill antigen bearing tumor cells
Source: J Immunother Cancer. 2022 Jun 21;10(6):e003752. doi: 10.1136/jitc-2021-003752 (PMC9214433; doi:10.1136/jitc-2021-003752)
Supplement: Supplementary data [file jitc-2021-003752supp001.pdf]

Supplementary Figure 1

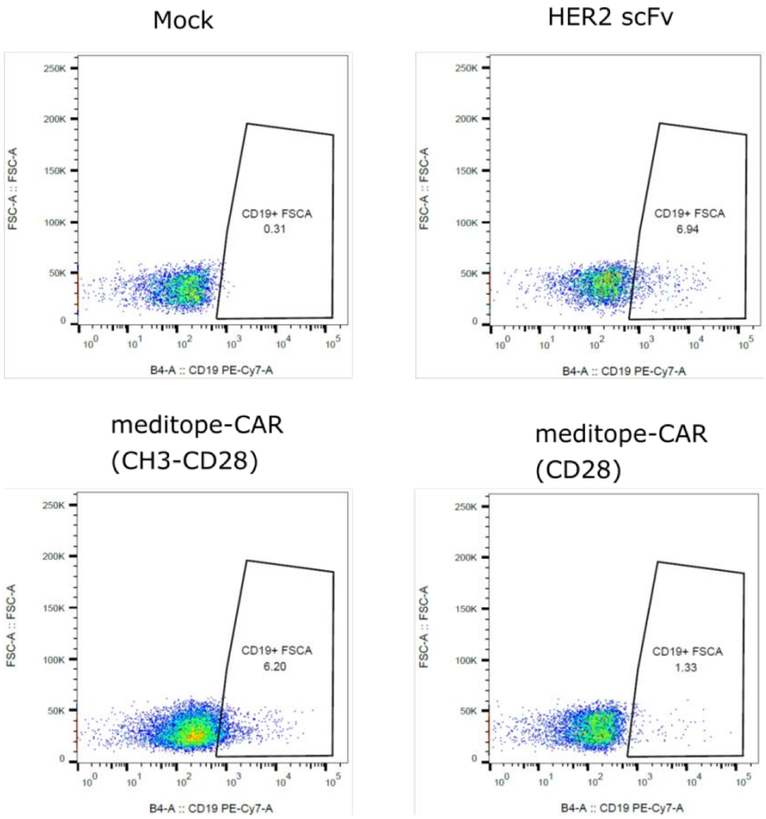

Fig. S1. CAR expression based on CD19t detection by flow cytometry. CHO-S cells were transiently transfected with indicated CAR. Mock represents that no plasmid was added during transfection.

Supplementary Figure 2

A

	Light chain				Heavy chain	
Kabat number	8 - - 11	38 - - - - - - - 46	83 - 85	99 - 101	39 - 41	87 - 89
Cetuximab	PVIL	QRTNGSPRL	IAD	GAG	QSP	TAI
Trastuzumab parental	PSSL	QKPGKAPRL	FAT	GQG	QAP	TAV
Trastuzumab memAb	PILL	QRTNGSPKL	EAD	GAG	QSP	TAI
αEGFR/HER3 parental	PSSL	QKPGKAPKL	FAT	GQG	QAP	TAV
αEGFR/HER3 memAb	PILL	QRTNGSPRL	EAD	GQG	QSP	TAI
αCDH6 parental	PSSL	QKPGKAPKL	FAT	GQG	QAP	TAV
αCDH6 memAb	PILL	QRTNGSPRL	FAD	GAG	QAP	TAI
Lintuzumab parental	PSSL	QKPGKAPKL	FAT	GQG	QAP	TAV
Lintuzumab memAb	PSIL	QRTNKAPRL	EAD	GAG	QSP	TAI

B

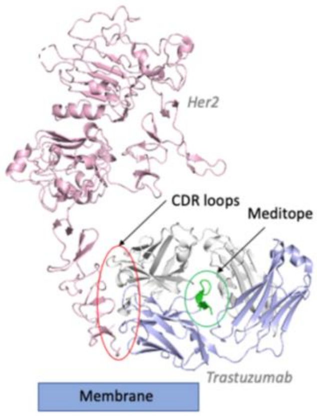

Fig. S2. The sequence difference between parental and corresponding meditope-enabled antibody. (A) To enable antibody to bind to meditope, we make mutations shown in red color. **(B)** Superposition of our meditope enabled trastuzumab (PDB: 4ioi) and parental trastuzumab bound to Her2 (PDB: 1n8z). The CDR loops are circled in red and the meditope binding site is circled in green.

Supplementary Figure 3

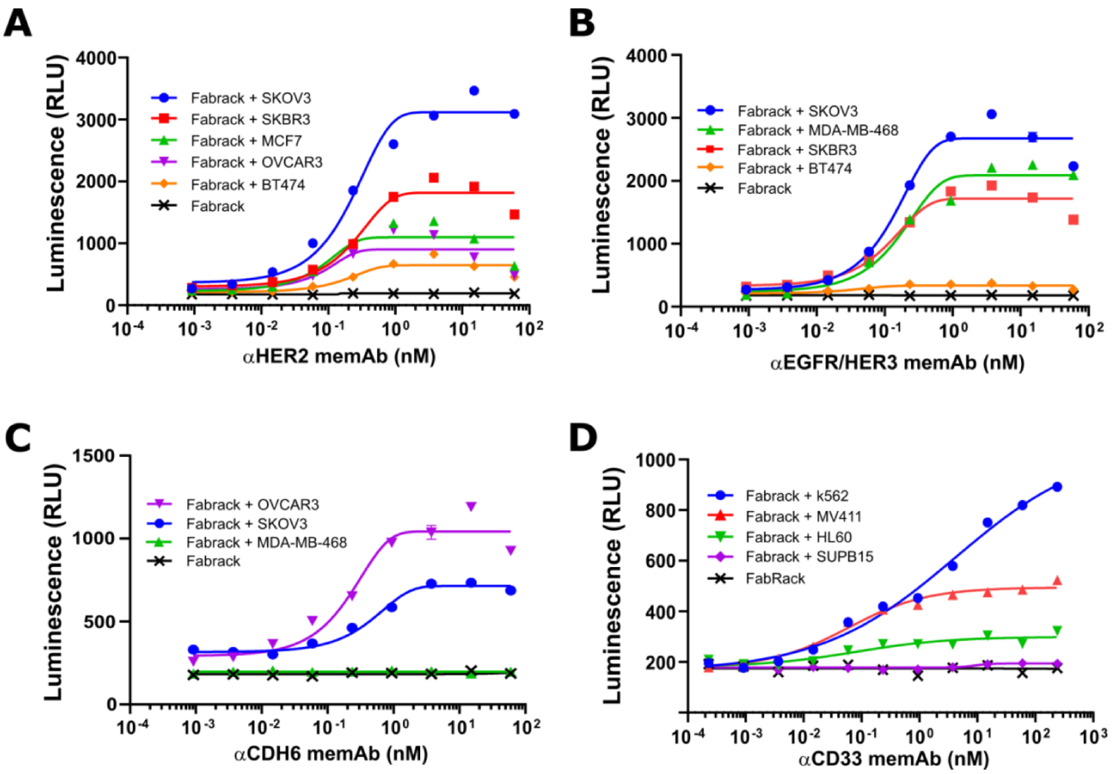

Fig. S3. Activation of Fabrack Jurkat cells (CH3-41BB) in the presence of target cells and a memAb. Fabrack Jurkat cell activation based on NFAT-regulated luciferase expression was examined after different concentrations of αHER2 memAb (**A**), αEGFR/HER3 memAb (**B**), αCDH6 memAb (**C**), or αCD33 memAb **(D)** were used to redirect Fabrack Jurkat cells to target cells. Experiments were done in technical duplicates. (Mean ± SEM)

Supplementary Figure 4

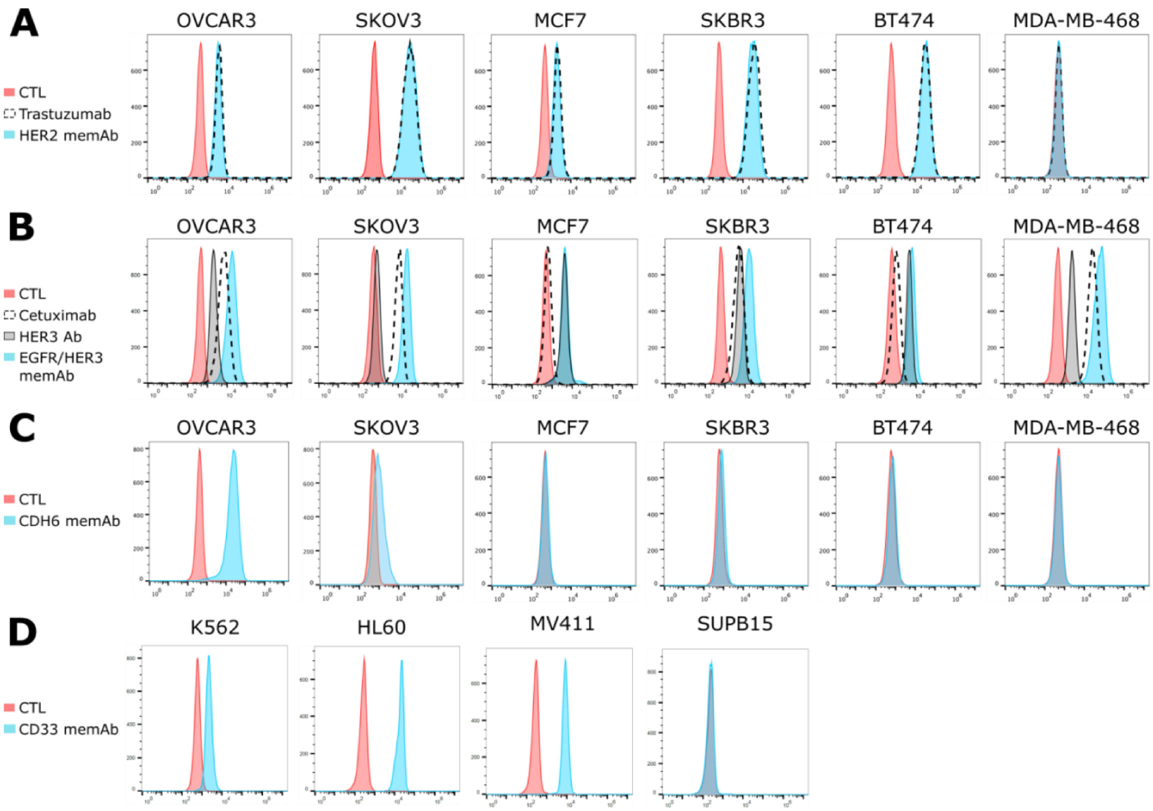

Fig. S4. Validation of memAb binding to target cancer cell lines. HER2 (**A**), EGFR/Her3 (**B**), CDH6 (**C**) or CD33 (**D**) expression at cell surface of each cancer cell line was detected by indicated memAb. Trastuzumab, cetuximab and a commercial HER3 antibody were included for comparison. OVCAR3 and SKOV3 are human ovarian cancer cell lines. MCF7, SKBR3, BT474 and MDA-MB-468 are human breast cancer cell lines. K562, HL60, MV411 and SUPB15 are human leukemia cancer cell lines.

Supplementary Figure 5

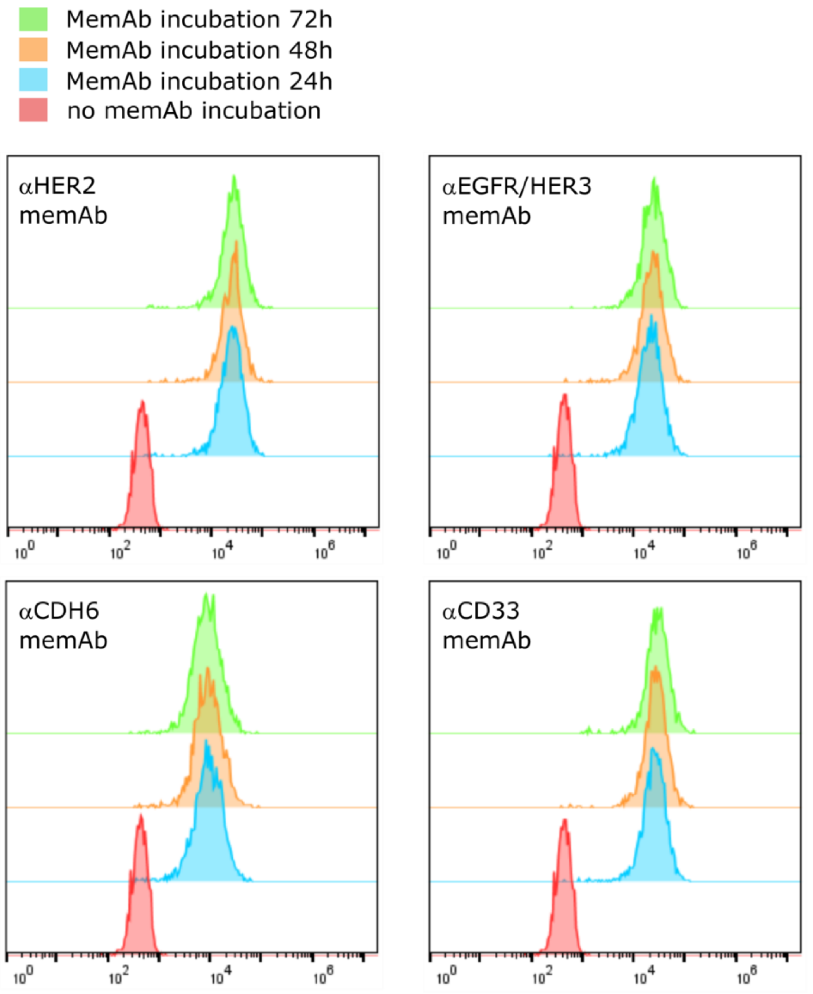

Fig. S5. The continuous existence of Fabrack CAR on Jurkat cells after memAb incubation. Fabrack Jurkat cells were incubated with 100 nM indicated memAb for 24, 48 or 72h in the absence of target tumor cells. Cells were stained with secondary anti-human kappa-Alexa-647 to confirm continuous memAb binding to the cell surface, which was indicative of no Fabrack internalization.

Supplementary Figure 6

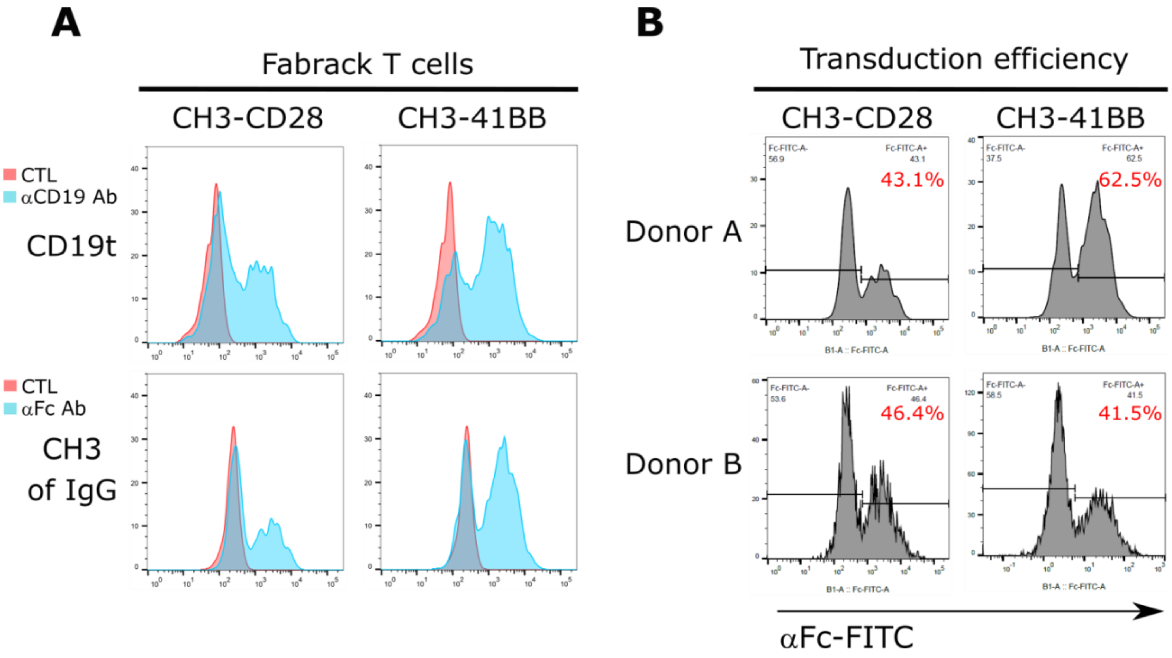

Fig. S6. Fabrack T cells validated for CAR expression at T cell surface. (**A**)Expression of Fabrack CAR CH3-CD28 or CH3-41BB construct at T cell surface was determined by staining cells with αCD19 (BD #557835) and αFc (Jackson # 109-606-008) antibodies in order to detect truncated CD19 and CH3 domain of IgG, respectively. **(B)** The transduction efficiency was mostly ~40% and reached highest to ~60% after T cells were transduced with lentivirus at MOI of 1.

Supplementary Figure 7

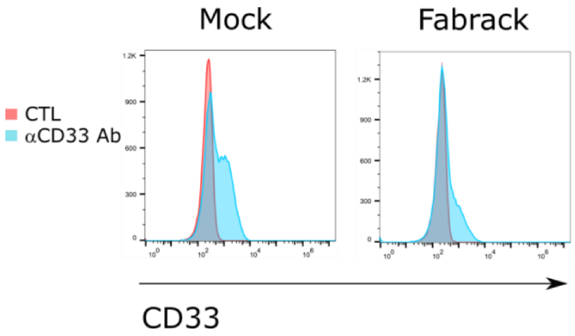

Fig. S7. CD33 expression at the mock and Fabrack T cell surface. Native human T cells transduced with or without Fabrack CAR (CH3-CD28) were stained with αCD33-PE antibody followed by flow cytometry analysis.

Supplementary Figure 8

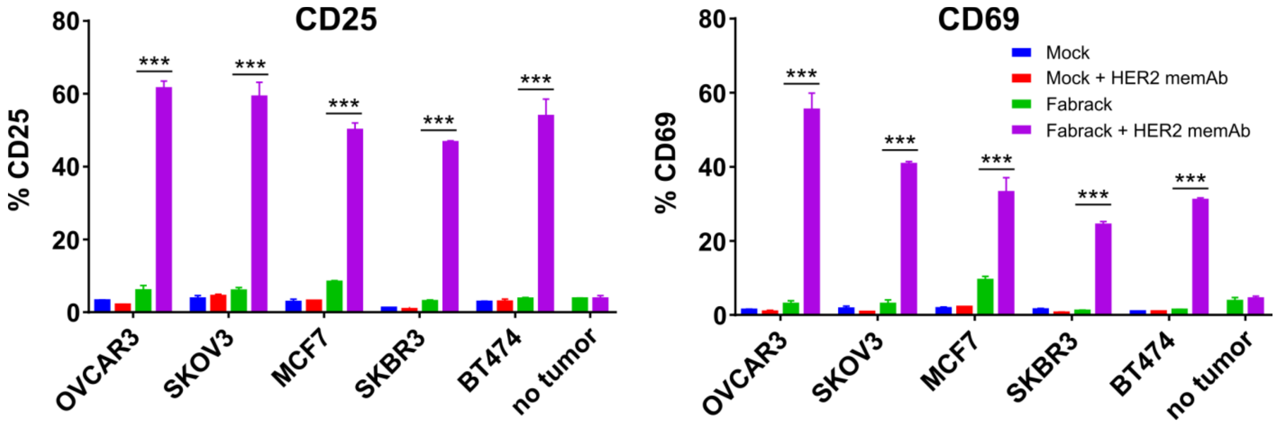

Fig. S8. Fabrack T cells validated for increased expression of CD25 and CD69 after incubation with target cells and a corresponding memAb. The percentage of T cells with increased CD25 or CD69 was shown. Increased expression of CD25 (left) and CD69 (right) was observed in Fabrack T cells after αHER2 memAb redirected Fabrack T cells to ovarian (OVCAR3 and SKOV3) or breast (MCF7, SKBR3 and BT474) cancer cells. Cells were incubated with or without 0.5 nM αHER2 memAb for 5 hours at a 1:1 E:T ratio. Activation markers at Fabrack T cell suface were analyzed by flow cytometry after staining cells with fluorescent dye-conjugated antibodies. Mock was gated on all CD3+ cells and Fabrack conditions were gated on CD19+(CAR+) cells. Experiments were done in technical duplicates. (Mean ± SEM, *** = P $\leq$ 0.001)

Supplementary Figure 9

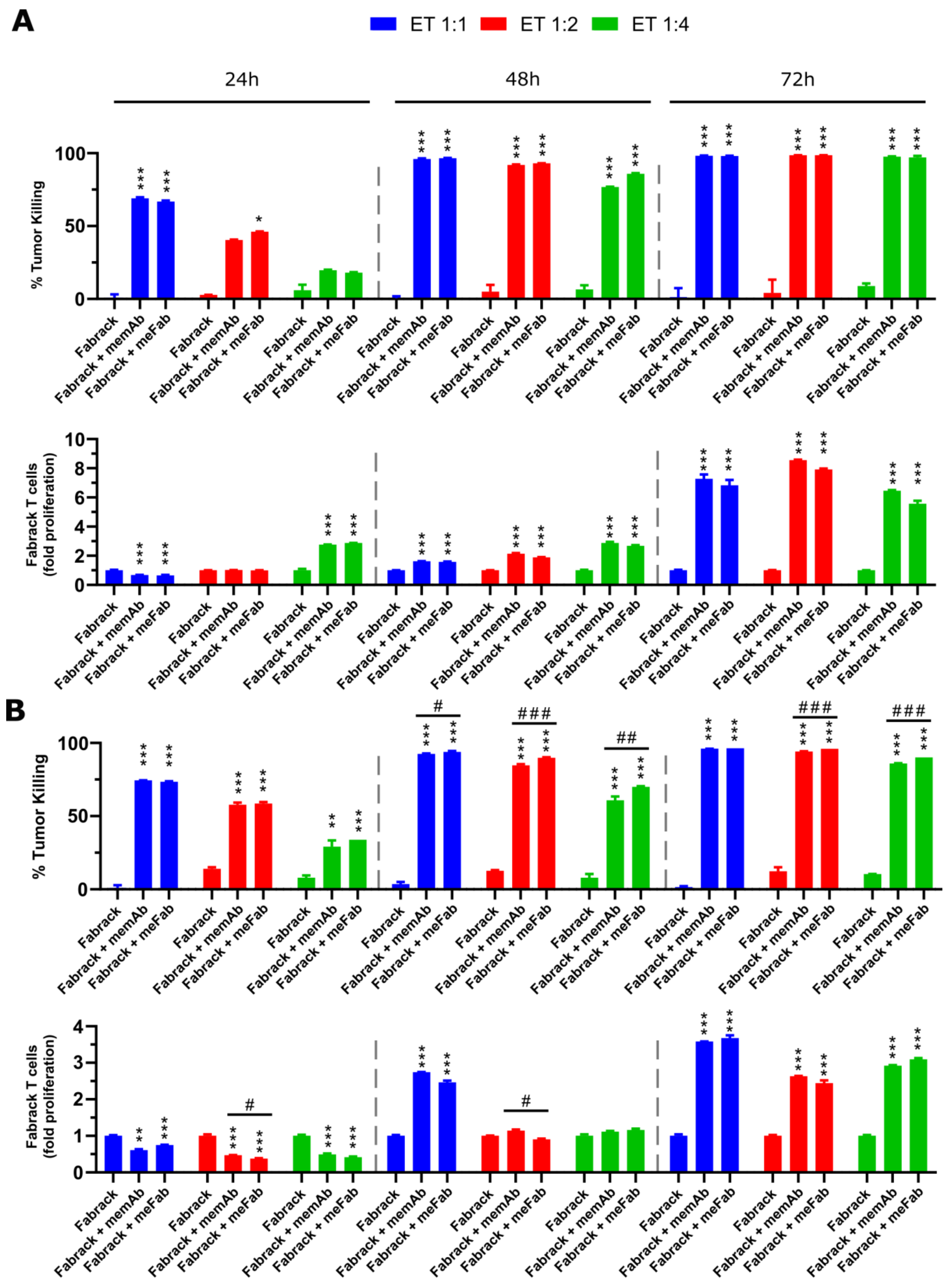

Fig. S9. Tumor killing and T cell proliferation after Fabrack T cells directed by HER2 memAb or meFab to target tumor. OVCAR3 cells (**A**) or SKBR3 cells (**B**) were treated with 0.5 nM HER2 memAb or meFab at indicated ET ratio for 24, 48 or 72h. The tumor killing and T cell proliferation were analyzed by flow cytometry. Experiments were done in technical duplicates. Killing was based on tumor counts co-cocultured with Mock T cells. "#" indicates significance between memAb and meFab treated cells. "*" indicates significance versus Fabrack treated cells. (Mean ± SEM, # and * = P $\leq$ 0.05, ## and ** = P $\leq$ 0.01, ### and *** = P $\leq$ 0.001)

Supplementary Figure 10

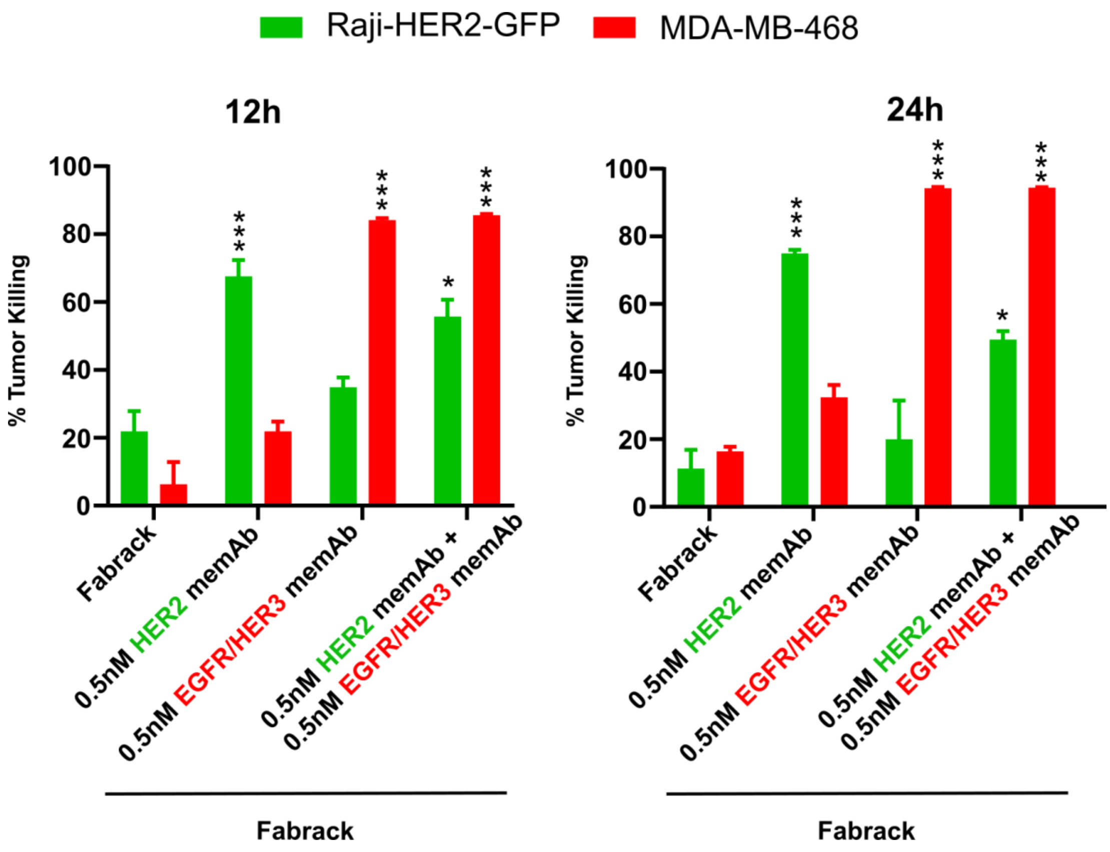

Fig. S10. MAb-specific killing of Raji-HER2-GFP and MDA-MB-468 by Fabrack T cells. To analyze the mAb-specific killing, 1.25 × 10^4 Raji-HER2-GFP cells and 1.25 × 10^4 MDA-MB-468 were seeded in a well of 96-well round bottom plate and treated by Fabrack T cells at ET ratio 1:2 with indicated memAb. After 12h or 24h incubation, cells were harvested, stained, and further analyzed by flow cytometry. Killing was based on tumor counts co-cocultured with Mock T cells. Experiments were done in technical duplicates. (Mean ± SEM, * = P $\leq$ 0.05, *** = P $\leq$ 0.001)

Supplementary Figure 11

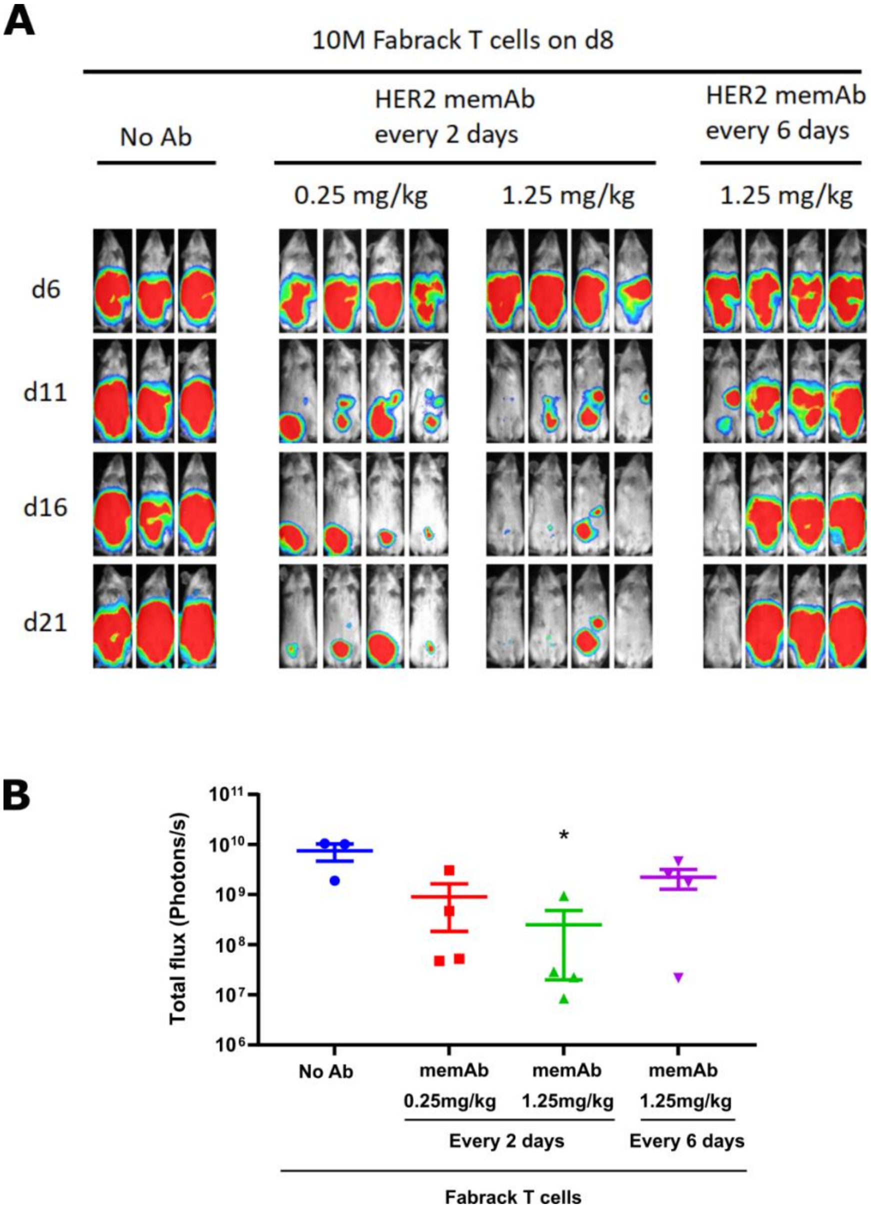

Fig. S11. The effect of antibody dosing regimen combined with Fabrack T cells on tumor suppression. **(A)** Mouse images of OVCAR3 tumor burden was shown on day 6, 11, 16 and 21 after tumor injection on day 1. Mice treated with 1.25 mg/kg HER2 memAb every two days (total 7 doses) had greater tumor suppression than other groups. **(B)** Tumor burden of mice in different treatment groups was plotted based on total flux on day 21. (n=3 or 4, mean ± SEM, * = P $\leq$ 0.05)

Supplementary Figure 12

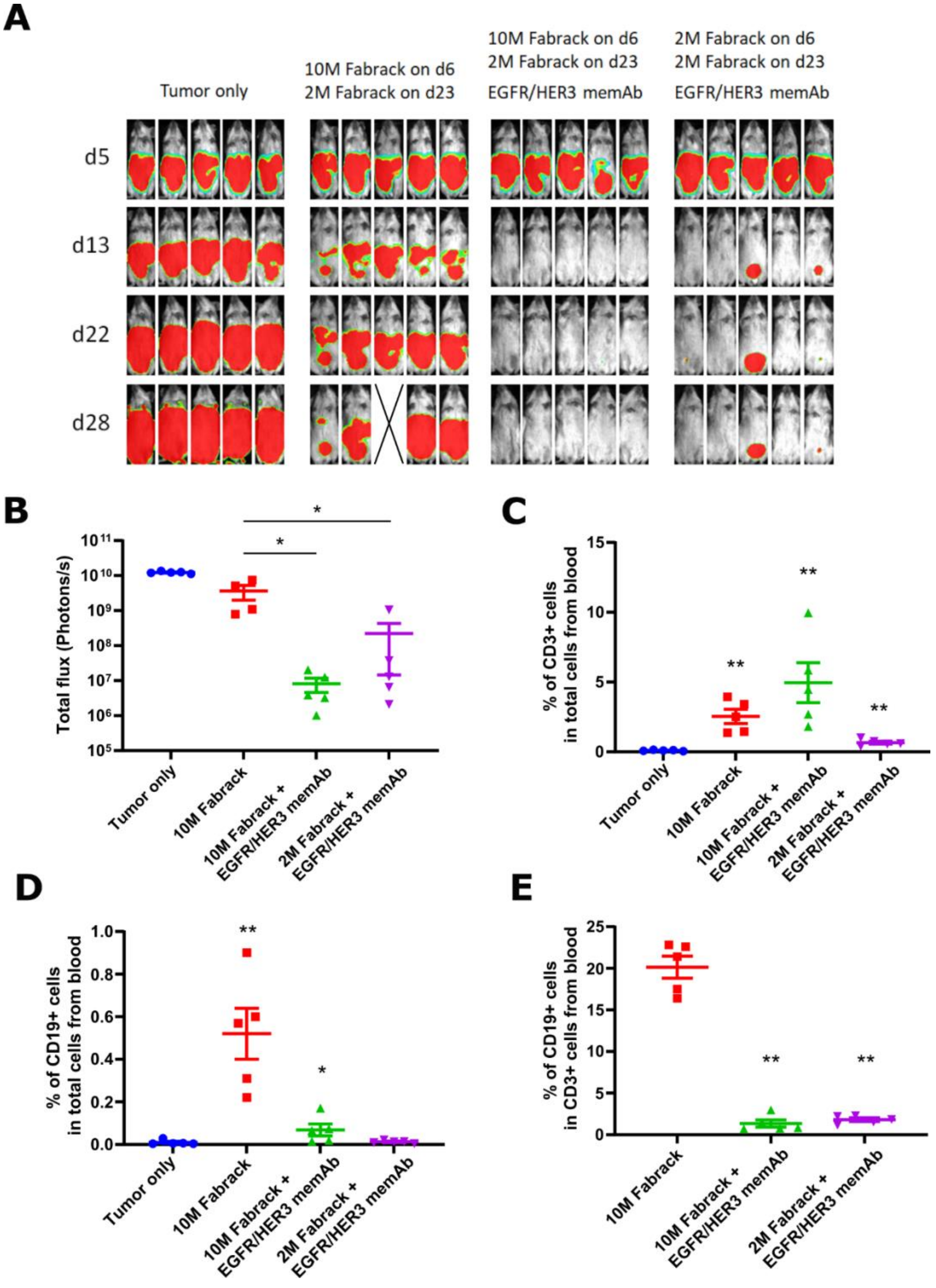

Fig. S12 The effect of high and low dose of Fabrack T cells on OVCAR3 xenograft tumor model. (A) Mouse images of OVCAR3 tumor burden were shown on day 5, 13, 22 and 28. Mice were intraperitoneally given first dose of 10M or 2M Fabrack T cells on day 6 and second dose of 2M Fabrack T cells on day 23. EGFR/HER3 memAb at a dose of 1.25mg/kg was intraperitoneally given on day 5, 8, 11, 14, 17, 22 and 25. **(B)** Tumor burden of mice in different treatment groups was plotted based on total flux on day 28. (n=4 or 5, mean ± SEM**,** * = P $\leq$ 0.05) **(C)** The percentage of human CD3+ T cells in mouse blood cells on day 20 was analyzed by flow cytometry. "*" indicates the significance versus tumor only group. (n=5, mean ± SEM, ** = P $\leq$ 0.01) **(D)** The percentage of human CD19+ Fabrack T cells in mouse blood cells on day 20 was analyzed by flow cytometry. "*" indicates the significance versus tumor only group. (n=5, mean ± SEM, * = P $\leq$ 0.05, ** = P $\leq$ 0.01) **(E)** The percentage of human CD19+ Fabrack T cells in human CD3+ cells from mouse blood on day 20 was analyzed by flow cytometry. "*" indicates the significance versus Fabrack treated group. (n=5, mean ± SEM, ** = P $\leq$ 0.01)

Supplementary Table 1

Antigen (Antibody)	Median Fluorescence Intensity (MFI)					
	OVCAR3	SKOV3	MCF7	SKBR3	BT474	MDA-MB-468
Anti-Kappa as CTL (Abcam #ab202832)	453	558	496	627	622	492
HER2 (clinical trastuzumab)	3997	33035	2165	34826	30957	525
HER2 (HER2 memAb)	3582	34545	1964	28892	32635	510
EGFR (clinical cetuximab)	6588	10509	625	4722	1021	26422
HER3 (R&D #FAB3481R)	2070	854	4096	5761	4534	2536
EGFR + HER3 (EGFR/HER3 memAb)	16495	25474	4248	15584	6025	61997
CDH6 (CDH6 memAb)	11034	961	512	787	717	502

Table S1. The level of HER2, EGFR, HER3 or CDH6 expression in ovarian or breast cancer cell lines. Antibodies indicated in the first column were used to detect antigen expression in ovarian (OVCAR3 and SKOV3) or breast (MCF7, SKBR3, BT474 and MDA-MB-468) cancer cell lines. Median fluorescence intensity was obtained from the flow cytometry data shown in supplementary figure 4.

Supplementary Table 2

Antigen (Antibody)	Median Fluorescence Intensity (MFI)			
	K562	MV411	HL60	SupB15
Anti-Fc as CTL (Abcam #98596)	602	286	280	242
CD33 (CD33 memAb)	2245	8405	20540	249

Table S2. The level of CD33 expression in leukemia cancer cell lines. Antibodies indicated in the first column were used to detect CD33 expression in K562, MV411, HL60 and SUPB15 cancer cell lines. Median fluorescence intensity was obtained from the flow cytometry data shown in supplementary figure 4.
